# Hypothalamic loss of *Snord116* recapitulates the hyperphagia of Prader-Willi syndrome

**DOI:** 10.1172/JCI97007

**Published:** 2018-01-29

**Authors:** Joseph Polex-Wolf, Brian Y.H. Lam, Rachel Larder, John Tadross, Debra Rimmington, Fàtima Bosch, Verónica Jiménez Cenzano, Eduard Ayuso, Marcella K.L. Ma, Kara Rainbow, Anthony P. Coll, Stephen O’Rahilly, Giles S.H. Yeo

**Affiliations:** 1 Medical Research Council (MRC) Metabolic Diseases Unit, University of Cambridge Metabolic Research Laboratories, Wellcome Trust–MRC Institute of Metabolic Science, Addenbrooke’s Hospital, Cambridge, United Kingdom.; 2 Center of Animal Biotechnology and Gene Therapy and Department of Biochemistry and Molecular Biology, School of Veterinary Medicine, Universitat Autònoma de Barcelona, Bellaterra, Spain, and Centro de Investigación Biomédica en Red de Diabetes y Enfermedades Metabólicas Asociadas (CIBERDEM), Barcelona, Spain.

**Keywords:** Genetics, Metabolism, Obesity

## Abstract

Profound hyperphagia is a major disabling feature of Prader-Willi syndrome (PWS). Characterization of the mechanisms that underlie PWS-associated hyperphagia has been slowed by the paucity of animal models with increased food intake or obesity. Mice with a microdeletion encompassing the *Snord116* cluster of noncoding RNAs encoded within the Prader-Willi minimal deletion critical region have previously been reported to show growth retardation and hyperphagia. Here, consistent with previous reports, we observed growth retardation in *Snord116^+/–P^* mice with a congenital paternal *Snord116* deletion. However, these mice neither displayed increased food intake nor had reduced hypothalamic expression of the proprotein convertase 1 gene *PCSK1* or its upstream regulator *NHLH2*, which have recently been suggested to be key mediators of PWS pathogenesis. Specifically, we disrupted *Snord116* expression in the mediobasal hypothalamus in *Snord116^fl^* mice via bilateral stereotaxic injections of a Cre-expressing adeno-associated virus (AAV). While the Cre-injected mice had no change in measured energy expenditure, they became hyperphagic between 9 and 10 weeks after injection, with a subset of animals developing marked obesity. In conclusion, we show that selective disruption of *Snord116* expression in the mediobasal hypothalamus models the hyperphagia of PWS.

## Introduction

Prader-Willi syndrome (PWS) causes severe obesity, with affected individuals displaying profound food-seeking behavior (1, 2). People affected by PWS progress along a complex natural history with distinct phases, commencing with an early “failure to thrive” characterized by hypotonia and feeding difficulties. Hyperphagia, decreased energy expenditure, and obesity appear later in childhood (3). PWS is typically associated with large deletions on human chromosome 15; however, recent patient reports have shown that microdeletions encompassing the *SNORD116* cluster of noncoding RNAs (ncRNAs) on paternal chromosome 15q11.2 result in a phenotype that overlaps substantially with PWS (4–8). These patients display most of the major diagnostic features of PWS (9, 10), thereby suggesting a PWS minimal deletion region encompassing *SNORD116* and 2 neighboring ncRNAs, RNAs *IPW* and *SNORD109A* (Supplemental Figure 1; supplemental material available online with this article; https://doi.org/10.1172/JCI97007DS1).

The *SNORD116* genomic locus encodes multiple species of ncRNAs, including long noncoding RNAs (lncRNAs), arrays of small nucleolar RNAs (snoRNAs), and lncRNAs with snoRNA ends (sno-lncRNAs), the function of which remains largely unknown (11–13). The Prader-Willi region on human chromosome 15 is syntenic to mouse chromosome 7, with the *SNORD116* cluster in both species comprising an imprinted array of snoRNA repeats interspersed by exons of an lncRNA host gene, *116HG* (12, 14). *SNORD116/Snord116* is also transcribed as part of a large transcriptional unit denoted as small nucleolar host gene 14 (*SNHG14*, formerly *LNCAT*) in both humans and mice. The progression of transcription through *SNHG14* is different between neuronal and nonneuronal cells (15–17), and in mice, expression of *Snord116* snoRNAs becomes enhanced within hypothalamic nuclei by weaning age (18).

None of the existing mouse models of PWS fully recapitulate the human phenotype (19). Many such models with gene deletions in the Prader-Willi region are lethal at the embryonic stage or have poor postnatal survival. Two viable mouse lines removing *Snord116* have been reported: a 150-kb deletion of the *Snord116* cluster from the paternal allele yielding the *Snord116*^+/–P^ mouse (20) and a deletion of *Snord116* with 2 exons of the neighboring ncRNA (21). In both models, there is no expression of any *Snord116* transcripts, as the maternal allele is silenced by imprinting. Mice with a maternal allele *Snord116* deletion are, unsurprisingly, indistinguishable from WT controls. Several additional *Snord116* deletion models have also been reported, including *Snord116* deletions that are biallelic (22), specific to neuropeptide Y (NPY) neurons (22), or tamoxifen inducible (23).

All *Snord116* deletion mouse models reported thus far remain smaller than controls through adulthood and do not transition to obesity as seen in human PWS — a summary of *Snord116* deletion model phenotypes is presented in Supplemental Table 1, with focus on growth and energy homeostasis. The most consistent phenotype observed in *Snord116*-deficient mice is low body weight and early growth retardation, reminiscent of failure to thrive in PWS infants. Differences in body weight start emerging between P2 and P5, and body length and fat mass are also reported to be diminished. A number of studies also report that *Snord116^+/–P^* mice appear to be hyperphagic.

Recently Burnett et al. have reported a human model of PWS using human iPS cell–derived (iPSC-derived) neurons (24). They report that such neurons, with deletions encompassing *Snord116*, show reduced expression of *PCSK1* (encoding prohormone covertase), which has been previously implicated in human obesity (25), and a transcription factor *NHLH2*, which regulates expression of the *PCSK1* gene (26, 27).

In the present study, we assessed energy intake phenotypes in the congenital *Snord116^+/–P^* mouse and found that differences in food intake are not significant when corrected appropriately for the effect of lower body weight. Deletion of *Snord116* specifically in the mediobasal hypothalamus of adult mice, however, revealed hyperphagia in the absence of early effects on growth. Of note, a subset of such animals developed marked obesity.

## Results

### Congenital paternal Snord116 deletion (Snord116^+/–P^) mice have impaired growth, but do not develop hyperphagia or obesity.

**Figure 1:**
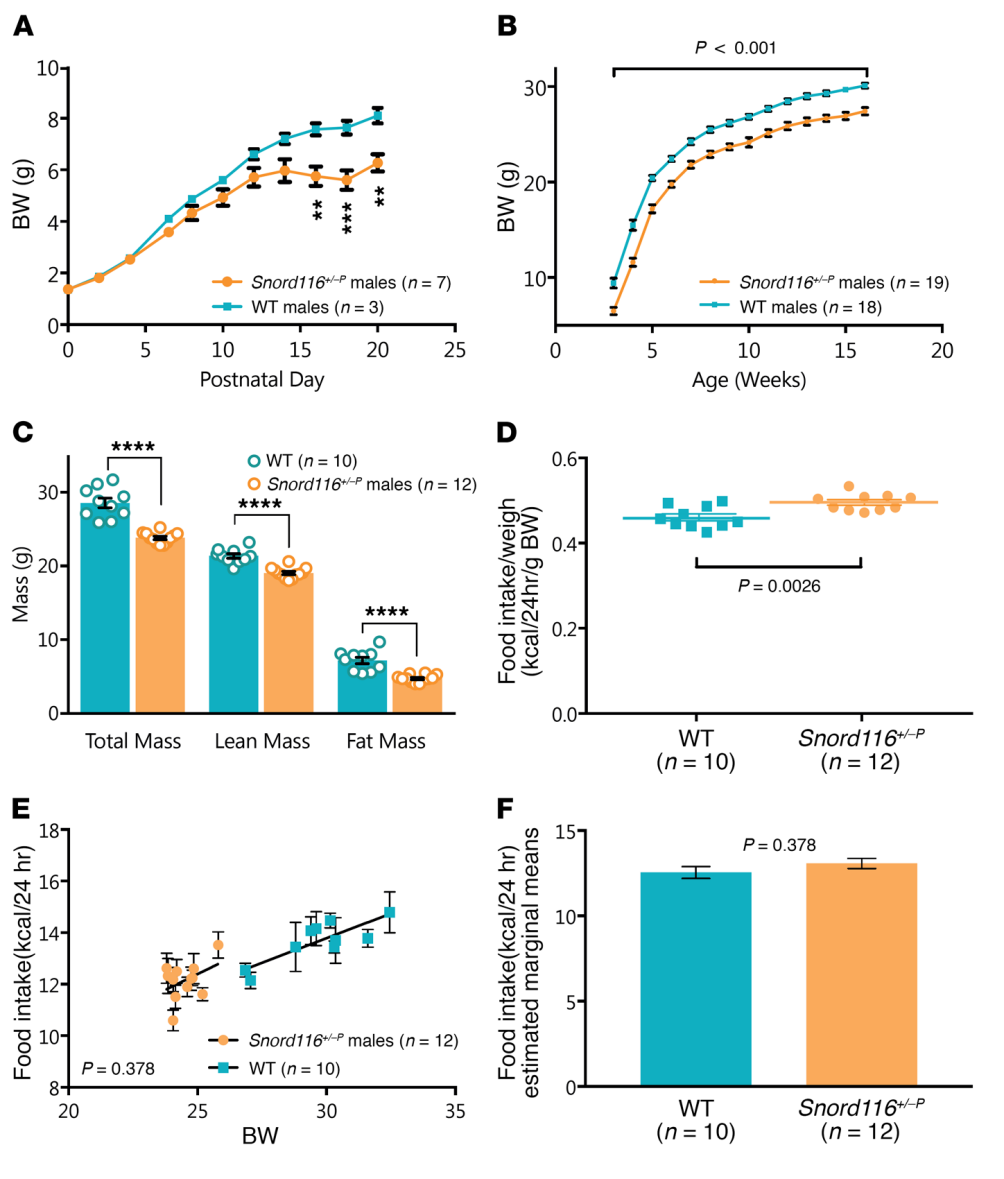
Congenital *Snord116* deletion mice display a growth phenotype, but do not transition to obesity or hyperphagia. (**A**) Postnatal body weight is lower in *Snord116^+/–P^* (*n* = 7) versus WT (*n* = 3) mice, measured to 20 days after birth. Significant weight differences were observed from day 16. **P < 0.01; ***P < 0.001, 2-way repeated-measures ANOVA. (**B**) Adult body weight in *Snord116^+/–P^* (*n* = 19) mice remains below that of WT (*n* = 18) mice, measured between 3 and 16 weeks of age, with significant effect of genotype on body weight observed throughout adulthood. *P* < 0.001, 2-factor mixed-design ANOVA. (**C**) All mass subcomponents are decreased in *Snord116^+/–P^* (*n* = 12) versus WT (*n* = 10) mice, as measured in adult males 13 to 16 weeks of age. ****P < 0.0001, *t* test. (**D**–**F**) Analysis of daily food intake (10-day average) in *Snord116^+/–P^* (*n* = 12) versus WT (*n* = 10) mice at 13–16 weeks of age reveals that, (**D**) when divided by body weight, food intake is significantly higher in *Snord116^+/–P^* mice (*t* test, *P* = 0.0026); however, when food intake is (**E**) plotted in relation to body weight as a covariate and (**F**) ANCOVA-corrected for differences in body weight, there is no evidence for altered food intake between WT and *Snord116^+/–P^* mice (ANCOVA, *P* = 0.378). Data are reported for male mice as mean ± SEM.

We investigated growth, body composition, and food intake phenotypes in mice with a congenital paternal deletion of *Snord116* (*Snord116^+/–P^*). As previously reported, *Snord116^+/–P^* mice had significantly lower body weight than WT littermates from early postnatal life (P16), which continued though adulthood (Figure 1, A and B). Similar results were also observed in female mice (Supplemental Figure 2). *Snord116^+/–P^* mice thus remain small without transitioning to developing obesity. Adult *Snord116^+/–P^* mice had reduced lean and fat mass as assessed by dual-energy x-ray absorptiometry (DEXA) (Figure 1C).

We next determined whether congenital *Snord116* deficiency may affect food consumption in adult mice. However, differences in body size needed to be taken into account. Previous reports accounted for these differences by dividing food intake by body weight (20, 22, 28, 29). When daily food intake measurements over a 10-day period were divided by total body weight in our study, we observed, as others had previously, significantly increased food intake in the *Snord116^+/–P^* mice (Figure 1D). The raw food intake data are shown in Supplemental Figure 3.

Considering that there is an inherent difference in body weight between *Snord116^+/–P^* and WT mice, we hypothesized that it is possible that the lower body weight of the *Snord116^+/–P^* mice may account for the relative differences in food intake. An improved method, based on analysis of covariance (ANCOVA), has been reported to better model energy homeostasis phenotypes under these circumstances (30). This allows us to test whether a difference in food intake exists when we remove the confounding effect of body weight. *Snord116^+/–P^* mice did not have significantly different 24-hour food intake compared with WT mice after differences in body weight were accounted for using this model (ANCOVA, *P* = 0.378) (Figure 1, E and F).

We also studied food intake after a 24-hour fast in *Snord116^+/–P^* versus WT mice. Using ANCOVA to correct for body-weight differences, there were no differences in food intake after either 4 hours (*P* = 0.714, ANCOVA) or 24 hours (*P* = 0.856, ANCOVA) after reintroduction of food (Supplemental Figure 4).

### Expression of leptin/melanocortin pathway genes in the hypothalamus of Snord116^+/–P^ mice across fed and food-deprived states remains normal.

**Figure 2:**
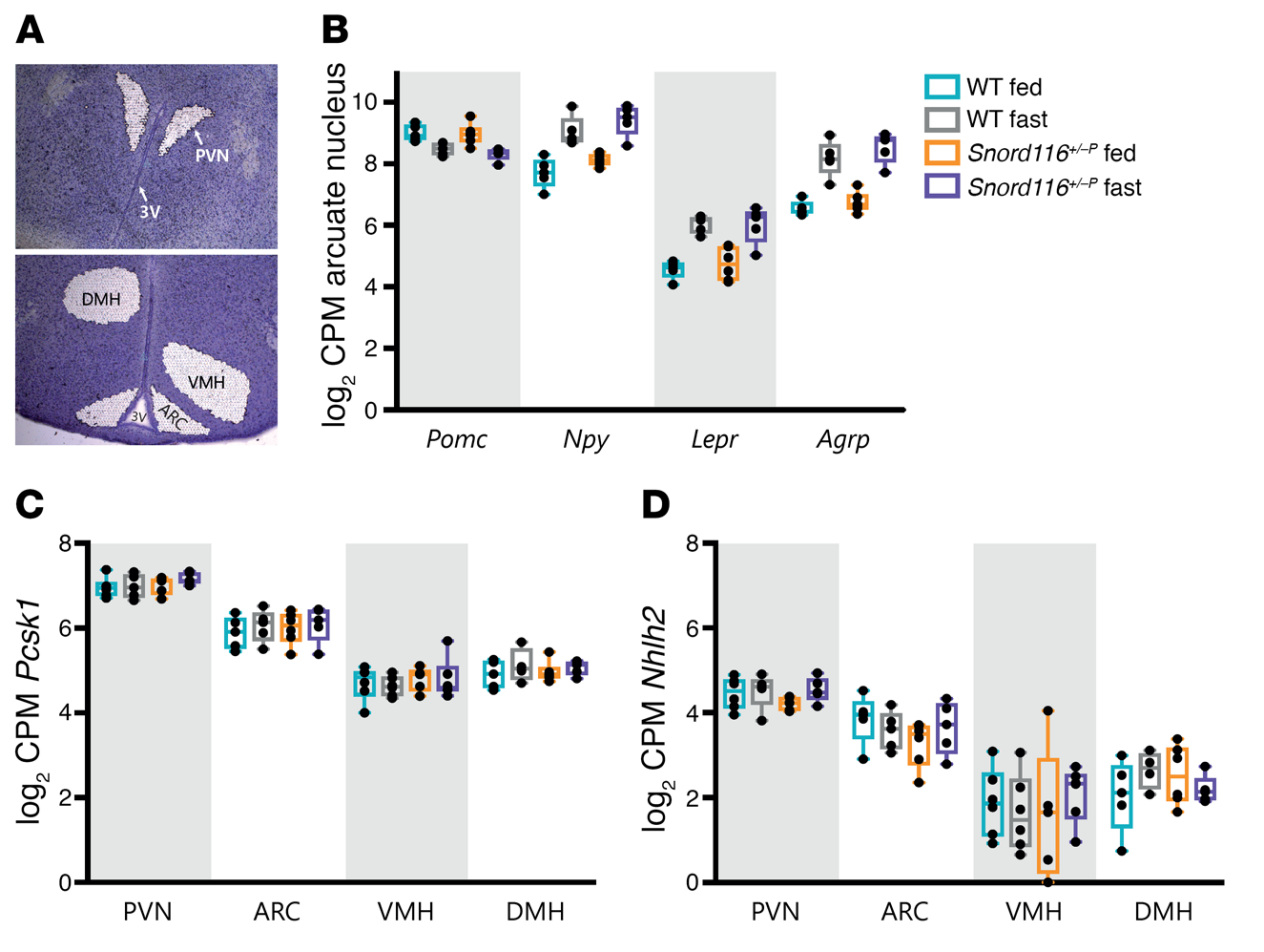
Expression of hypothalamic leptin/melanocortin pathway genes is unaltered in congenital *Snord116* deletion compared with that in WT mice in both the ad libitum–fed and 24 hour–fasted states. (**A**) Representative image of laser-captured nuclei from mouse hypothalamus (cresyl violet stained). Original magnification, ×50. (**B**) The nutritionally regulated expression pattern of *Pomc*, *Npy*, *Lepr*, and *Agrp* does not change in congenital *Snord116* deletion mice in the ARC. Genotype by nutritional state interaction for the 3 genes is not significant. *P* > 0.6, FDR = 1, Benjamini-Hochberg FDR procedure. Expression of (**C**) *Pcsk1* and (**D**) *Nhlh2* does not significantly differ by genotype or nutritional state (ad libitum fed versus 24 hour fasted) across 4 different laser-captured hypothalamic nuclei. For **B**–**D**, expression is shown for male mice 16–19 weeks of age (*n* = 4–6 mice per condition) as log_2_ counts per million (CPM), with box plots showing the median, interquartile range, and extrema.

To assess whether *Snord116* deficiency affects the expression of hypothalamic genes in appetitive pathways, we evaluated expression of leptin/melanocortin pathway genes. RNA-sequencing (RNA-seq) was performed using RNA extracted from laser-captured hypothalamic nuclei from adult (16 to 19 weeks old) WT and *Snord116^+/–P^* mice in either the ad libitum–fed or 24-hour fasted states (Figure 2A). We verified that laser-capture microdissection yielded material specific to the dissected nucleus by verifying enriched expression of positive control genes (Supplemental Figure 5). Within the arcuate nucleus (ARC), the expression of *Npy*, leptin receptor (*Lepr*), and agouti-related protein (*Agrp*) showed the expected increase with fasting in both WT and *Snord116^+/–P^* mice (FDR < 0.03). Proopiomelanocortin (*Pomc*) also displayed a trend for a decreased expression with fasting in both WT (log_2_ fold change [FC] = –0.52, *P* < 0.03, FDR = 0.53) and *Snord116^+/–P^* (log_2_ FC = –0.67, *P* < 0.003, FDR = 0.23) mice (Figure 2B). Following from recent work suggesting that *Snord116* deficiency disrupts expression of prohormone convertase 1 (*Pcsk1*) and its transcription activator, nescient helix-loop-helix 2 (*Nhlh2*) (24), we examined hypothalamic expression of both genes in *Snord116^+/–P^* mice. *Pcsk1* and *Nhlh2* mRNA were detectable in all 4 hypothalamic nuclei. No differences in expression were observed by genotype or nutritional status within any of the 4 nuclei (paraventricular nucleus [PVN], ARC, ventromedial hypothalamus [VMH], or dorsomedial hypothalamus [DMH]) (Figure 2, C and D). These data were confirmed in RNA extracts from whole hypothalami obtained from a different set of experimental animals, which also demonstrated that there was no evidence of differential expression of *Pcsk1* and *Nhlh2* between WT and *Snord116^+/–P^* mice in either fed or fasted states (Supplemental Figure 6).

### Adult-onset deletion of Snord116 in the mediobasal hypothalamus results in hyperphagia, with a subset of mice further developing obesity and increased fat mass.

**Figure 3:**
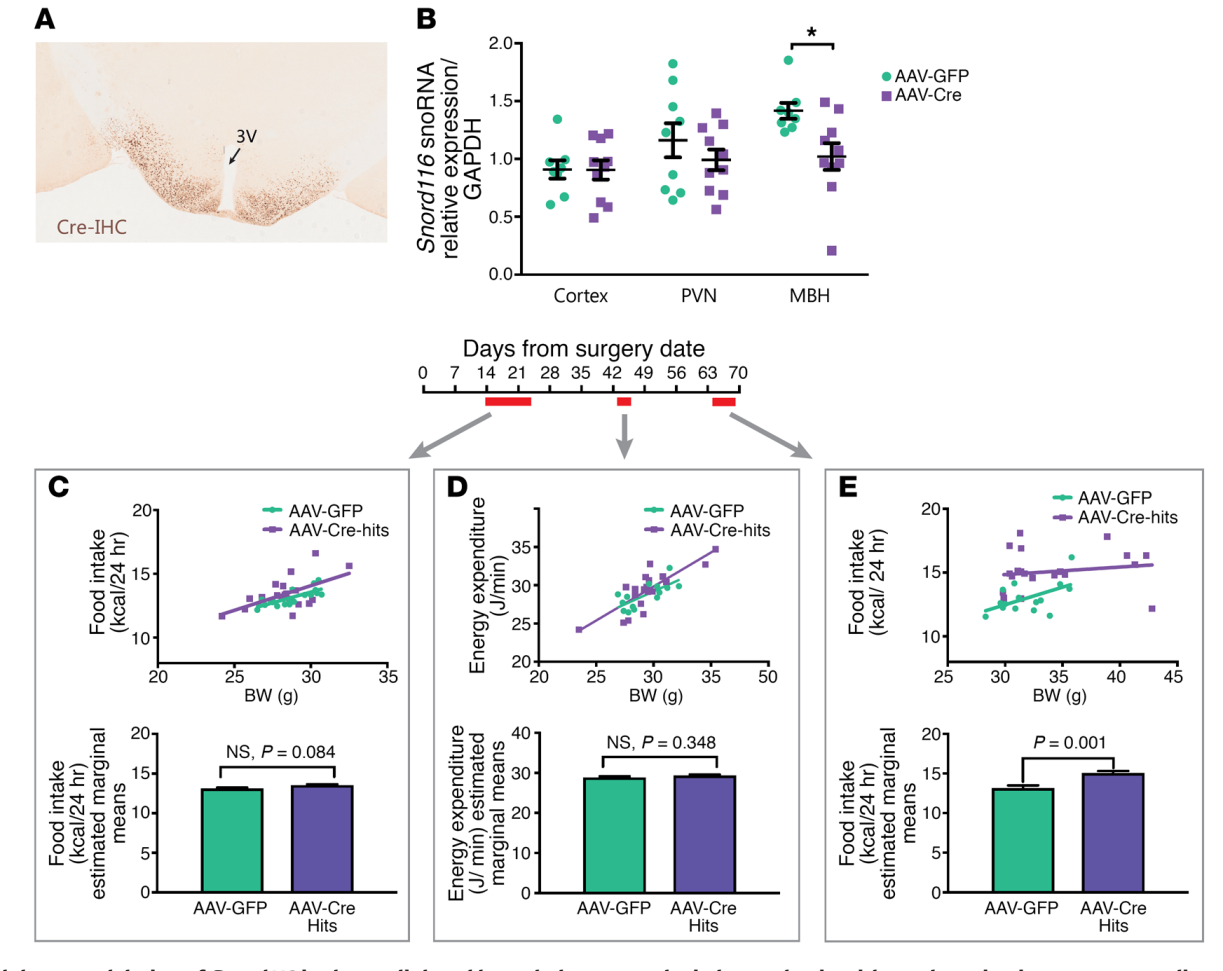
Adult-onset deletion of *Snord116* in the mediobasal hypothalamus results in hyperphagia with no alteration in energy expenditure. (**A**) Bilateral targeting of the mediobasal hypothalamus was performed with AAV-Cre injection and verified with Cre immunohistochemistry. 3V, third ventricle. Original magnification, ×200. (**B**) Micropunches of 3 brain regions revealed a selective decrease of *Snord116* expression in the mediobasal hypothalamus in AAV-Cre–injected (*n* = 10) mice (28% decrease, *P* = 0.01, *t* test) compared with AAV-GFP controls (*n* = 9). Bars display mean ± SEM. (**C**) Ten-day average food intake measured from 2 weeks after stereotaxic surgery was not significantly different between AAV-GFP– and AAV-Cre–treated mice (*P* = 0.084, ANCOVA). (**D**) No differences in energy expenditure were observed over 72-hour indirect calorimetry commencing 6 weeks after viral delivery (*P* = 0.348, ANCOVA). (**E**) Significantly higher food intake (corrected for the covariate of body weight) was observed in AAV-Cre–treated mice versus AAV-GFP–treated controls (*P* = 0.001, ANCOVA) when measured over 5 days commencing 9 weeks after viral delivery. For **C**–**E**, top panels display scatter plots of body weight versus either food intake or energy expenditure and bottom panels display food intake/energy expenditure values that are ANCOVA-corrected for body weight (estimated marginal mean ± SEM). All data are reported for male mice, with surgery commencing at 10 to 12 weeks of age.

An adult-onset mediobasal hypothalamic deletion of *Snord116* was generated by stereotaxic injection of AAV-Cre (where AAV indicates adeno-associated virus) into *Snord116^fl^* mice at 10 weeks of age, with AAV-GFP–injected mice used as controls. Successful bilateral targeting of Cre for each mouse was verified using Cre immunohistochemistry (Figure 3A). Of 26 AAV-Cre injections, 21 were verified as hits, with bilateral expression of Cre in the mediobasal hypothalamus, while 5 were excluded from analysis as misses, with no bilateral hypothalamic expression of Cre. Cre expression in all hits and misses is documented in Supplemental Figure 7. Efficacy of the Cre-mediated deletion of *Snord116* was verified in a separate set of AAV-Cre–injected (*n* = 10) and AAV-GFP–injected (*n* = 9) mice, with a reduction of *Snord116* expression in the mediobasal hypothalamus of Cre-injected mice (28%, *P* = 0.01, Student’s *t* test), but not in other regions, including the PVN and cerebral cortex (Figure 3B).

Food intake and energy expenditure were assessed in the postsurgery period. Food intake was measured over 2 time points: first, between 2 and 4 weeks after surgery, when no weight differences were evident between the Cre- versus GFP-injected mice, and second, between 9 and 10 weeks after surgery, when some Cre-injected mice showed increased body weight compared with controls. Energy expenditure was measured at 6 weeks after surgery, when differences in weight between the Cre- and GFP-injected mice began to appear. There were no detectable differences between Cre- and GFP-injected mice when measured between 2 and 4 weeks after surgery (*P* = 0.084, ANCOVA) (Figure 3C). Differences in energy expenditure as measured through indirect calorimetry were not significant when assessed at 6 weeks after surgery (*P* = 0.348, ANCOVA) (Figure 3D). Food intake studies performed between 9 and 10 weeks after surgery revealed consistent hyperphagia in Cre-injected mice regardless of body weight (*P* = 0.001, ANCOVA) (Figure 3E).

**Figure 4:**
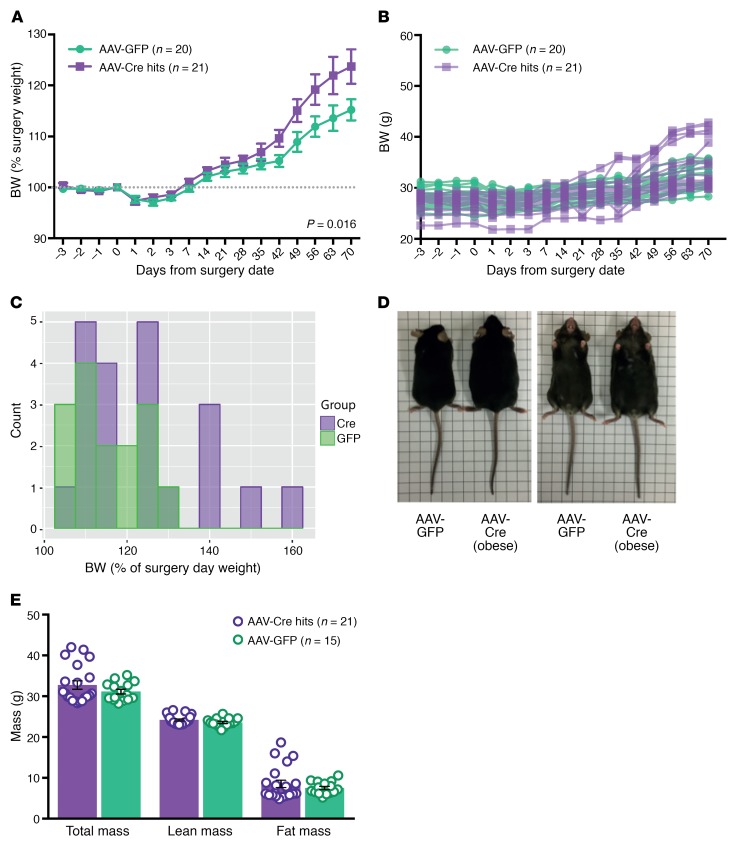
A subset of Cre-targeted mice develop obesity concomitant with increased fat mass. (**A**) Percentage of body-weight change after stereotaxic surgery of AAV-GFP and AAV-Cre into the mediobasal hypothalamus of *Snord116^fl^* male mice (mean ± SEM). AAV-Cre–treated mice had a greater increase in body weight than AAV-GFP–treated controls. *P* = 0.016, 2 way repeated-measures mixed ANOVA. (**B**) Individual body-weight curves of AAV-Cre–injected mice (*n* = 21) compared with AAV-GFP–injected mice (*n* = 20). (**C**) Body-weight histogram of mice at 10 weeks after stereotaxic injection. (**D**) Comparison of AAV-GFP–treated control mouse with AAV-Cre–treated mouse from the obese subset. (**E**) Body composition as measured with DEXA in AAV-Cre– and AAV-GFP–treated mice at 10 weeks after surgery (mean ± SEM).

Body weight and composition were also assessed over the 10-week postsurgery period. AAV-Cre–injected mice gained more weight (as a percentage of presurgery weight) compared with AAV-GFP–injected controls (*P* = 0.016, ANOVA), with differences appearing from 6 weeks after surgery (Figure 4A). Analysis of the individual body-weight curves highlighted greater variability in the weight gain of AAV-Cre–injected mice (final weight, 29.8–42.8 g) compared with AAV-GFP–injected mice (final weight, 28.8–35.8 g) (Figure 4B), and at 10 weeks after surgery, a subset (5/21) of AAV-Cre–treated mice gained over 140% of presurgery weight (Figure 4C). Mice in this obese AAV-Cre–treated subset appeared obviously larger than AAV-GFP controls (Figure 4D). Body composition was analyzed (DEXA) at 10 weeks after surgery, with increased fat mass underlying the obesity of a subset of Cre-treated mice (Figure 4E and Supplemental Figure 8).

### Snord116^fl^ Cre-injected mice have normal hypothalamic leptin-melanocortin gene expression, with obese animals displaying increased Socs3 expression.

**Figure 5:**
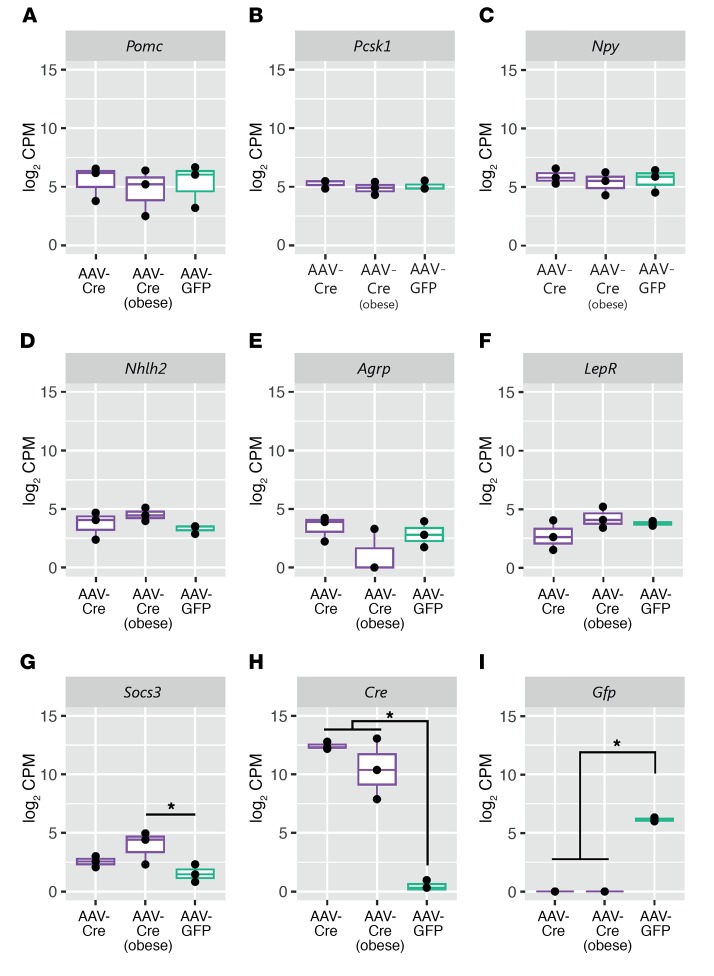
Cre-targeted mice have unaltered hypothalamic leptin-melanocortin gene expression, but increased *Socs3* expression in obese animals. Box plots of gene expression from laser-captured hypothalamic sections from AAV-Cre hits with less than 25% body-weight gain after surgery (AAV-Cre, *n* = 3); AAV-Cre hits with more than 40% body-weight gain after surgery (AAV-Cre obese, *n* = 3); and AAV-GFP controls (*n* = 3). (**A**–**F**) Expression of *Pomc*, *Pcsk1*, *Npy*, *Nhlh2*, *Agrp*, and *LepR* is unchanged between the AAV-Cre–treated groups and AAV-GFP controls. (**G**) Expression of hypothalamic *Socs3* is increased in AAV-Cre obese mice (FDR = 0.08), and (**H**) expression of Cre (FDR = 2.37 × 10^–45^) and (**I**) GFP (FDR = 5.55 × 10^-22^) are only present within the respective treatment groups. Data shown are from mice at 10 weeks after surgery with expression as log_2_ counts per million. FDR values were generated using the Benjamini-Hochberg FDR procedure.

We analyzed whether the hyperphagic phenotype observed in the AAV-Cre–targeted mice may be driven by altered expression of hypothalamic genes in appetitive pathways. RNA-seq was performed on laser-captured hypothalamic regions obtained from adjacent brain sections with Cre immunohistochemistry as well as matching regions from AAV-GFP–injected mice. For the AAV-Cre mice, we analyzed gene expression in mice that were from the obese (>140% presurgery body weight) and nonobese subsets. No significant changes were detected in the expression of *Pomc*, *Lepr*, *Npy*, *Agrp*, *Pcksk1*, or *Nhlh2* (for all genes, *FDR* > 0.38) (Figure 5, A–F). In obese AAV-Cre–treated mice compared with AAV-GFP–injected controls, we observed increased expression of suppressor of cytokine signaling 3 (*Socs3*) (FDR = 0.08) (Figure 5G), a gene that has been suggested to affect hypothalamic signaling pathways downstream of leptin (31, 32). *Cre* and *Gfp* expression was, as expected, observed only in the samples of those specific treatment groups (Figure 5, H and I). Complete lists of differentially expressed genes are provided in Supplemental Table 2.

We further assayed serum collected at 10 weeks after surgery to assess physiological changes that may accompany adult hypothalamic *Snord116* deletion. A small but significant difference in total thyroxine (T4) was observed (*P* = 0.005, Mann-Whitney *U* test) in all AAV-Cre hits compared with that in GFP-injected controls (Supplemental Figure 9A). No significant differences were detected in levels of testosterone or IGF-1 (Supplemental Figure 9, B and C).

## Discussion

Small deletions encompassing the *SNORD116* cluster result in a phenotype substantially overlapping with PWS in humans, commencing with an early failure to thrive and with a later onset of hyperphagia and obesity. Limited aspects of the PWS phenotype have, however, been recapitulated with deletions of *Snord116* in mice.

Consistent with previous reports (20, 22, 28), we observed impaired growth in congenital *Snord116^+/–P^* mice from early postnatal life and lower body weights throughout adulthood. Furthermore, we did not observe hyperphagia in the congenital *Snord116^+/–P^* mice using a previously reported ANCOVA-based method (30) to appropriately account for the low body weight of these mice.

Recent work suggests that defects in prohormone processing may underlie the appetitive phenotype in PWS, with *SNORD116* deficiency linked to decreased expression of *PCSK1* and its associated regulator *NHLH2* in patient-derived iPSCs as well as across several tissues in *Snord116^+/–P^* mice (24). We observed no differences in hypothalamic *Pcsk1* expression in *Snord116^+/–P^* mice in either ad libitum–fed or fasted states and also found no alterations in *Nhlh2* expression. Within the hypothalamus, PCSK1 is responsible for processing a number of substrates relevant to energy homeostasis control, and within the ARC, this interfaces with multiple components of the leptin/melanocortin pathway, including POMC, NPY, and AGRP in humans (25). Our findings indicate that the expression of these components of the arcuate leptin/melanocortin pathway, at least in mice, is not perturbed with congenital *Snord116* deficiency. We observed consistent upregulation of *Npy*, *Agrp,* and *Lepr* mRNA with fasting in the ARC of both WT and *Snord116^+/–P^* mice. *Npy* and *Agrp* upregulation in states of food deprivation has been extensively reviewed (33–35), and leptin receptor expression has been reported to increase as leptin levels fall during food restriction, occurring preferentially within *Agrp* rather than *Pomc* neurons (36, 37). Thus, it appears that *Snord116* is not essential for functional nutritionally regulated transcriptional responses of these genes in the ARC. Our results draw parallels to human findings highlighting the lack of aberrant leptin levels in PWS patients versus obese controls (after adolescence) or nonobese controls (prior to weight gain) (38–40).

Since mice with congenital *Snord116* deletions do not undergo the phenotypic transition seen in human PWS, we generated adult-onset *Snord116* deletions to determine whether phenotypes mirroring later stages of human PWS (i.e., obesity and hyperphagia) might appear in the absence of a developmental growth phenotype. An acute adult deletion also excludes the effect of compensatory changes that may occur as a result of congenital deletions, similar to that reported for *Agrp,* where a food-intake phenotype is evident only in adult, but not neonatal, mice (41). We observed that adult-onset deletion of *Snord116* results in a delayed hyperphagia, observed at 9 weeks after the stereotaxic surgery. The lag in manifesting the phenotype could be due to less efficient Cre-Lox recombination for larger regions (150 kb *Snord116* cluster) (42), which can require a longer period of exposure to Cre before sufficient recombination occurs.

While hyperphagia was found in the AAV-Cre–injected mice, there was variability in weight, with a subset of mice gaining more than 140% of their starting body weight. We demonstrate that Cre injection specifically diminishes *Snord116* expression in the mediobasal hypothalamus and sought to minimize the effects of technical variability by examining viral targeting to exclude animals where AAV-Cre was not delivered or was only detected unilaterally. Weight variability remained present, however, even when we excluded all animals in which virus was not expressed in the hypothalamus. While the variability in body weight seen may have a methodological explanation, other explanations are worthy of consideration. *Snord116* is a paternally expressed gene cluster, and aspects of the cluster may crosstalk with other imprinted genes as part of an imprinted gene network in neurons (43). Recent reports suggest that dysregulation of imprinted gene networks (such as that seen with insufficiency of another imprinted gene, *Trim28*) can lead to polyphenism in which both lean and obese phenotypes are observed despite the identical underlying genotype and a highly controlled environment.

The presence of hyperphagia, concomitant with obesity in a subset of mice, after *Snord116* deletion in the mediobasal hypothalamus of adults indicates that *Snord116* plays a role in the hypothalamic control of appetite and food intake. It further provides a murine model recapitulating aspects of the later stages of human PWS, specifically phases 2b and 3, in which weight begins to increase in parallel with increased appetite, followed by fully fledged hyperphagia (44). We thus propose that the developmental and energy homeostasis phenotypes of PWS are dissociable in the mouse and that deletion of *Snord116* can lead to robust hyperphagia in the absence of an early growth phenotype (presented in Supplemental Figure 10).

Interestingly, however, these mice seem to increase in weight due to hyperphagia without evident changes in energy expenditure. The hyperphagia and obesity in our mouse model further contrasts with findings from a tamoxifen-inducible model of *Snord116* deletion, in which there was no difference in body weight and reduced food intake (when corrected for body weight) (45). Considering that Cre-Lox recombination may be less efficient for the 150-kb *Snord116* cluster, a transient exposure to Cre with tamoxifen (compared with a sustained exposure to AAV-driven Cre expression) might account for some of these differences.

Similar to what was shown in our data from congenital *Snord116^+/–P^* mice, we did not detect any changes in the expression of *Pcsk1*, *Nhlh2*, or other leptin/melanocortin pathway genes in the mediobasal hypothalamus of the hyperphagic *Snord116^fl^* Cre-injected animals. This suggests that alternative explanations for the hyperphagia of *SNORD116* deficiency are required. Of interest, we observed that *Socs3* expression was significantly increased in the hypothalamus of obese AAV-Cre–treated mice compared with GFP controls (log_2_ FC = 2.7). While *Socs3* levels may increase as a result of obesity in these animals, this increase appears to be graded, with nonobese AAV-Cre animals showing a trend for increased hypothalamic *Socs3* expression (log_2_ FC = 1.2). *Socs3* is a negative regulator of leptin signaling and has been proposed to mediate central leptin resistance (31, 46), and mice with modestly overexpressed *Socs3* in POMC neurons have increased body weight and adiposity (47).

In conclusion, we report that adult deletion of *Snord116* in the mediobasal hypothalamus represents a hyperphagic murine model of PWS, with a subset of mice further developing obesity concomitant with increased fat mass. Our work highlights that modulating the onset of *Snord116* deletion in mice can recapitulate differing phenotypes from the natural history of human PWS. This provides a unique model for further studies investigating the pathophysiology of PWS as well as a platform for testing the efficacy of interventions aiming to prevent or reverse hyperphagia in PWS.

## Methods

### Animal husbandry, breeding, and genotyping

Animals were housed in a 12-hour light/12-hour dark cycle in a temperature-controlled (22°C) facility, with ad libitum access to food and water unless stated otherwise. Mice were weaned between P19 and P21 and were kept on RM3 expanded chow (Special Diet Services). All mice were on a C57BL/6J background, with *Snord116^+/–P^* (B6[Cg]-Snord116tm1.1Uta/J; JAX stock no. 008149) and *Snord116^fl^* mice (B6[Cg]-Snord116tm1Uta/J; JAX stock no 008118) obtained from Jackson Laboratories. Genotyping was performed as previously reported (20), with a mix of 3 primers (Sigma Aldrich): WT forward (AATCCCCAACCTACTTCAAACAGTC); deletion forward primer (TTTACGGTACATGACAGCACTCAAG); and common reverse primer (TGGATCTCTCCTTGCTTGTTTTCTC).

### Measurement of body mass and composition

For *Snord116^+/–P^* mice, postnatal body weight was measured in 3 independent mouse litters on scales calibrated to 0.01 g. For adult mice, measurements of body weight were performed once per week from 3 to 16 weeks of age on scales calibrated to 0.1 g. For the mice with *Snord116* deletion in mediobasal hypothalamus, body weight was measured daily from 3 days before to 3 days after stereotaxic surgery, after which body weight was measured weekly until 10 weeks after surgery on scales calibrated to 0.1 g. Body composition was measured using DEXA with a Lunar PIXImus Mouse Densitometer (GE Healthcare Systems). Prior to DEXA, mice were administered a terminal dose of anaesthetic (Dolethal 200 mg/ml solution, Vetoquinol UK Ltd.).

### Hypothalamic quantitative PCR

Quantitative PCR (qPCR) was performed on an ABI 7900 real-time PCR system on RNA extracted using an miRNeasy RNA Extraction Kit (QIAGEN) from microdissected mouse hypothalami from WT and *Snord116^+/–P^* mice using gene-specific primers for *Gapdh* (Mm99999915_g1, TaqMan, Thermo Fisher Scientific), *Pcsk1* (Mm00479023_m1, TaqMan, Thermo Fisher Scientific), and *Nhlh2* (Mm01959164_u1, TaqMan, Thermo Fisher Scientific).

### Measurement of food consumption and energy expenditure

Daily food intake was performed with single-housed mice using scales calibrated to 0.1 g. For the congenital deletion mice, food intake was measured over 10 days in mice between 13 and 15 weeks of age. *Snord116^fl^* mice undergoing stereotaxic surgery underwent 2 food-intake studies, a 10-day food-intake study at 2 weeks after surgery and a 5-day food-intake study at 9 weeks after surgery. Fast-refeeding studies were performed after a 24-hour fast, with food intake measured at 1, 2, 4, 8, 12, and 24 hours after reintroduction of chow or a 45% fat palatable high-fat, high-sugar (HFHS) diet (D12451, Research Diets). Metabolic rate measurements were made over a period of 72 hours, with animals single-housed in Techniplast cages. Animals were single housed 1 week prior to measurement for acclimatization, and energy expenditure was monitored on a custom-built system (Idea Studios). Oxygen consumption (VO_2_), carbon dioxide production (VCO_2_), respiratory exchange ratio (VCO_2_/VO_2_) (RER), and energy expenditure were assessed by the system over the course of the run at intervals of 10 to 15 minutes. Four cohorts of animals were used for the energy expenditure, each at between 5 and 6 weeks of the date of surgery. Energy expenditure (*EE*) in joules (*J*) at every time point was calculated based on indirect calorimetry using the Elia and Livesey equation: *EE*(*J*) = 15.818 × VO_2_ + 5.176 × VCO_2_ (48). Energy expenditure measurements in the first 3.5 hours at the start of the run were discarded in order for the animals to reach a steady state. Food intake and energy expenditure were corrected for body weight using ANCOVA (30), which was performed using the generalized linear model (glm) analysis in SPSS 23.0 (IBM) with body weight as a covariate.

### Stereotaxic surgery

Homozygous *Snord116^fl^* male mice at 10 to 12 weeks of age were injected with AAV viral vectors while under isoflurane anesthesia, similarly to the method described previously (49). Viral vectors used were either AAV7-CAG-oCre (3 × 10^13^ vg/ml) for test animals or AA7-CMV-GFP (2.1 × 10^12^ vg/ml). Vectors were generated by Fàtima Bosch’s group and utilized similarly to what was indicated in previous reports (50). Virus (200 nl) was injected bilaterally into the mediobasal hypothalamus, at 1.5 mm caudal to bregma (51), and plus or minus 0.28 mm laterally to the midline, and 6 mm below the skull. Injections were delivered with a 5 μl Hamilton syringe, with the needle left in place for 10 minutes after viral delivery to prevent reflux of the solution, and then gradually lifted in 2-minute steps at 4 mm and 2 mm below the skull surface.

### Verification of viral targeting

At 10 weeks after surgery, mice were terminally anesthetized with Dolethal and brains were excised, frozen on dry ice, and stored at –80°C. RNA expression levels of *Snord116* in brains of AAV-injected mice were tested using qPCR from 1 mm micropunches of the mediobasal hypothalamus obtained with a biopsy punch (Kai Medical), with micropunches of the PVN and the cortex used as controls. RNA was extracted using an miRNeasy RNA Extraction Kit (QIAGEN) from mouse tissue, and cDNA was generated using the Superscript VILO cDNA Synthesis Kit (Thermo Fisher Scientific). qPCR was then performed on an ABI 7900 Real-Time PCR System using the gene-specific primers for Snord116 snoRNA (Mm03455667_s1, TaqMan, ABI) and Gapdh (Mm99999915_g1, TaqMan, ABI). For all animals included in the metabolic analyses, the presence of Cre was tested for with immunohistochemistry on serial 20-μm sections of fresh-frozen brains. After mounting onto glass slides, tissues were fixed overnight in 10% formalin at room temperature. The following day, slides were washed once for 5 minutes in TBS, placed into a sealed container with 1× target-retrieval solution, pH 9 (Dako), and heated to 97°C for 40 minutes. After cooling to room temperature, endogenous peroxidase was blocked for 10 minutes, after which tissues were washed for 5 minutes in TBS and blocked in antibody diluent (1% BSA, 0.3% Triton-X in TBS) for 1 hour before incubating with a primary antibody overnight at 4°C (Novagen, catalog 69050, rabbit anti-Cre, 1:10,000). Slides were then washed 3 times for 10 minutes in 1× wash buffer (TBST, Dako) and incubated for 40 minutes at room temperature with a secondary antibody (HRP-labeled polymer anti-rabbit, EnVision+ System, Dako), followed by three 10-minute washes in TBST. DAB (DAKO Liquid DAB+ Substrate Chromogen System) was then applied for 3 minutes before a final wash in water, ethanol dehydration, xylene wash, and mounting. Imaging was performed at 20× using a Hamamatsu NanoZoomer S60 digital slide scanner. Successful targeting was defined by the presence of bilateral Cre expression within the mediobasal hypothalamic region (bregma –1.22 to –2.70). Presence of bilateral Cre was scored blindly by 2 researchers, and data for all samples are included in Supplemental Figure 7.

### Serum profiling

Serum samples were collected from AAV-injected mice via cardiac puncture from terminally anesthetized mice at 10 weeks after surgery. Total thyroxine (T4) was measured using a Dimension EXL autoanalyser (Siemens). IGF-1 was measured using the Quantikine Mouse IGF-1 ELISA Kit (R&D Systems), and testosterone was measured using the Testosterone Mouse ELISA Kit (Demeditec).

### Laser-capture microdissection

#### Snord116^+/–P^ fasted versus ad libitum–fed study.

Brains were collected from WT and *Snord116^+/–P^* mice between 17 and 19 weeks of age across 2 nutritional states, either ad libitum fed or after a 24-hour fast. Brains were fresh frozen and stored at –80°C before cryosectioning on a Bright OTF5000 cryostat. Consecutive 20-μm coronal sections were collected from 0.34 mm through 2.70 mm caudal to bregma (51) onto glass slides (Superfrost Plus, Thermo Fisher Scientific). Fresh-frozen brain sections were fixed in 95% ethanol (30 seconds) and rehydrated in an ethanol series (75% ethanol, 50% ethanol) prior to staining with cresyl violet (Ambion). Sections were then dehydrated in another ethanol series of 50%, 75%, 95%, and 100% ethanol and left to air dry prior to laser capture. Four nuclei (PVN, ARC, VMH, DMH) were then dissected from an average of 90 serial sections covering the mediobasal hypothalamus in mouse from bregma –0.34 to –2.70 mm using a PALM Microbeam Laser Capture Microdissection System (Zeiss) on a ×5 objective. Tissues were captured on AdhesiveCap Clear PCR tubes (Zeiss) and stored in 80 μl of QIAzol (QIAGEN) on dry ice prior to RNA extraction. Laser capture and subsequent cDNA library preparation were performed in 3 batches of 8 brains. The laser-capture procedure was similar to those used in previous reports (52, 53). RNA from laser-captured nuclei was extracted using the miRNeasy Micro RNA Extraction Kit (QIAGEN). Samples were then run on an Agilent 2100 Bioanalyzer using Agilent RNA 600 pico and/or nano chips to determine quantity and quality, with a typical sample RNA concentration in the range of 1 to 5 ng/μl.

#### AAV-injected Snord116^fl^ hypothalami.

Laser capture was performed using the same method as described above on single fresh-frozen brain slices, with hypothalamic regions excised based on the pattern of Cre immunohistochemistry in adjacent sections. Six AAV-Cre–injected mice were used, 3 with body-weight gain of greater than 140% of the presurgery weight (obese) and 3 with body-weight gain of less than 125%, as well as 3 region-matched AAV-GFP controls. Quantity and quality were assessed as above, with a typical sample RNA concentration between 70 and 200 pg/μl.

### Library preparation and RNA-seq

#### Snord116^+/–P^ fasted versus ad libitum–fed study.

Total RNA of 2 ng from each hypothalamic nucleus was SPIA amplified with an Ovation RNA-Seq System V2 (NuGEN). cDNA was fragmented to 200 bp using a Bioruptor Sonicator (Diagenode), followed by end repair and adapter ligation with the Encore Rapid DR Multiplex 1-96 Protocol to allow multiplex sequencing. Libraries of cDNA were quantified (KAPA Quantification Kit) and then submitted to CRUK Cambridge Institute (Cambridge, United Kingdom) for sequencing on an Illumina Hi-Seq 2500 in 2 to 3 lanes. Single-end reads (SE50) from 93 laser-captured hypothalamic nuclei were obtained, with an average of 15.63 million reads sequenced per sample.

#### AAV-injected Snord116^fl^ hypothalami.

Total RNA of 500 pg from each sample was used to prepare cDNA libraries using the Pico Input Mammalian SMARTer Stranded RNA-Seq Kit (Takara Bio). cDNA libraries were quantified on an Agilent 2100 Bioanalyzer using the high-sensitivity DNA kit (Agilent) and then submitted to CRUK-Cambridge Institute for sequencing on an Illumina Hi-Seq 4000 in 1 lane. Single-end reads (SE50) with an average of 9.02 million sequenced reads per sample were obtained.

### Bioinformatics

Reads were mapped to *Mus musculus* GRCm38 genome assembly (Ensembl) with TopHat, achieving an average mapping rate of 88.67% in the *Snord116^+/P^* study and 78.81% in the AAV injections study. Reproducible reads have been generated in this manner in orthogonal experiments (54–56). Analysis of RNA-seq data was performed in RStudio using the edgeR and limma packages. In the *Snord116^+/P^* study, outlier samples were detected based on global transcriptomic profiles in multidimensional scaling (MDS) plots using edgeR (57), with 8 of 93 samples excluded, so that there were 85 samples across 4 conditions (WT fast, WT fed, *Snord116^+/–P^* fast, *Snord116^+/–P^* fed), resulting in 4 to 6 biological replicates for each hypothalamic nucleus within each group. Transcriptomic data sets were deposited in the NCBI’s Gene Expression Omnibus database (GEO GSE96627, GSE102992, and GSE106210).

### Statistics

Graphs were generated in Graphpad Prism 6.0 or R, with details of the statistical tests used provided in the figure legends. Histogram of body weight 10 weeks after surgery was generated in R using the easyGgplot2 package. Differential gene expression was calculated using the glm functionality in EdgeR. SPSS 23.0 (IBM) was used to run ANOVA and ANCOVA tests. Unless otherwise noted, significance is defined at FDR < 0.1 and/or *P* < 0.05.

### Study approval

All mouse studies were performed in accordance with UK Home Office Legislation regulated under the Animals (Scientific Procedures) Act 1986 Amendment, Regulations 2012, following ethical review by the University of Cambridge Animal Welfare and Ethical Review Body (AWERB).

## Author contributions

JPW, BYHL, GSHY, APC, and SOR designed the research studies. JPW, BYHL, RL, JT, DR, MKLM, and KR conducted the experiments. VJC, EA, and FB produced the viral vectors. JPW and BYHL performed data/bioinformatics analyses. JPW, BYHL, GSHY, APC, and SOR wrote the manuscript.

## Supplementary Material

Supplemental data

Supplemental Table 1

Supplemental Table 2

## Version 1 01/29/2018

Electronic publication

## Version 2 01/30/2018

Corrected typo in the introduction.

## Version 3 03/01/2018

Print issue publication

## References

[1] Cassidy SB, Schwartz S, Miller JL, Driscoll DJ (2012). Prader-Willi syndrome. Genet Med.

[2] Butler JV, Whittington JE, Holland AJ, Boer H, Clarke D, Webb T (2002). Prevalence of, and risk factors for, physical ill-health in people with Prader-Willi syndrome: a population-based study. Dev Med Child Neurol.

[3] Miller JL (2011). Nutritional phases in Prader-Willi syndrome. Am J Med Genet A.

[4] Sahoo T (2008). Prader-Willi phenotype caused by paternal deficiency for the HBII-85 C/D box small nucleolar RNA cluster. Nat Genet.

[5] Duker AL (2010). Paternally inherited microdeletion at 15q11.2 confirms a significant role for the SNORD116 C/D box snoRNA cluster in Prader-Willi syndrome. Eur J Hum Genet.

[6] de Smith AJ (2009). A deletion of the HBII-85 class of small nucleolar RNAs (snoRNAs) is associated with hyperphagia, obesity and hypogonadism. Hum Mol Genet.

[7] Bieth E (2015). Highly restricted deletion of the SNORD116 region is implicated in Prader-Willi syndrome. Eur J Hum Genet.

[8] Anderlid BM, Lundin J, Malmgren H, Lehtihet M, Nordgren A (2014). Small mosaic deletion encompassing the snoRNAs and SNURF-SNRPN results in an atypical Prader-Willi syndrome phenotype. Am J Med Genet A.

[9] Holm VA (1993). Prader-Willi syndrome: consensus diagnostic criteria. Pediatrics.

[10] Gunay-Aygun M, Schwartz S, Heeger S, O’Riordan MA, Cassidy SB (2001). The changing purpose of Prader-Willi syndrome clinical diagnostic criteria and proposed revised criteria. Pediatrics.

[11] Yin QF (2012). Long noncoding RNAs with snoRNA ends. Mol Cell.

[12] Powell WT (2013). A Prader-Willi locus lncRNA cloud modulates diurnal genes and energy expenditure. Hum Mol Genet.

[13] Cavaillé J (2000). Identification of brain-specific and imprinted small nucleolar RNA genes exhibiting an unusual genomic organization. Proc Natl Acad Sci U S A.

[14] Vitali P, Royo H, Marty V, Bortolin-Cavaillé ML, Cavaillé J (2010). Long nuclear-retained non-coding RNAs and allele-specific higher-order chromatin organization at imprinted snoRNA gene arrays. J Cell Sci.

[15] Chamberlain SJ (2013). RNAs of the human chromosome 15q11-q13 imprinted region. Wiley Interdiscip Rev RNA.

[16] Leung KN, Vallero RO, DuBose AJ, Resnick JL, LaSalle JM (2009). Imprinting regulates mammalian snoRNA-encoding chromatin decondensation and neuronal nucleolar size. Hum Mol Genet.

[17] Martins-Taylor K (2014). Imprinted expression of UBE3A in non-neuronal cells from a Prader-Willi syndrome patient with an atypical deletion. Hum Mol Genet.

[18] Zhang Q, Bouma GJ, McClellan K, Tobet S (2012). Hypothalamic expression of snoRNA Snord116 is consistent with a link to the hyperphagia and obesity symptoms of Prader-Willi syndrome. Int J Dev Neurosci.

[19] Resnick JL, Nicholls RD, Wevrick R, Prader-Willi Syndrome Animal Models Working Group (2013). Recommendations for the investigation of animal models of Prader-Willi syndrome. Mamm Genome.

[20] Ding F (2008). SnoRNA Snord116 (Pwcr1/MBII-85) deletion causes growth deficiency and hyperphagia in mice. PLoS ONE.

[21] Skryabin BV (2007). Deletion of the MBII-85 snoRNA gene cluster in mice results in postnatal growth retardation. PLoS Genet.

[22] Qi Y (2016). Snord116 is critical in the regulation of food intake and body weight. Sci Rep.

[23] Purtell L, Qi Y, Campbell L, Sainsbury A, Herzog H (2017). Adult-onset deletion of the Prader-Willi syndrome susceptibility gene Snord116 in mice results in reduced feeding and increased fat mass. Transl Pediatr.

[24] Burnett LC (2017). Deficiency in prohormone convertase PC1 impairs prohormone processing in Prader-Willi syndrome. J Clin Invest.

[25] Stijnen P, Ramos-Molina B, O’Rahilly S, Creemers JW (2016). PCSK1 mutations and human endocrinopathies: from obesity to gastrointestinal disorders. Endocr Rev.

[26] Fox DL, Good DJ (2008). Nescient helix-loop-helix 2 interacts with signal transducer and activator of transcription 3 to regulate transcription of prohormone convertase 1/3. Mol Endocrinol.

[27] Jing E, Nillni EA, Sanchez VC, Stuart RC, Good DJ (2004). Deletion of the Nhlh2 transcription factor decreases the levels of the anorexigenic peptides alpha melanocyte-stimulating hormone and thyrotropin-releasing hormone and implicates prohormone convertases I and II in obesity. Endocrinology.

[28] Lin D (2014). Abnormal response to the anorexic effect of GHS-R inhibitors and exenatide in male Snord116 deletion mouse model for Prader-Willi syndrome. Endocrinology.

[29] Ding F, Li HH, Li J, Myers RM, Francke U (2010). Neonatal maternal deprivation response and developmental changes in gene expression revealed by hypothalamic gene expression profiling in mice. PLoS ONE.

[30] Tschöp MH (2011). A guide to analysis of mouse energy metabolism. Nat Methods.

[31] Wunderlich CM, Hövelmeyer N, Wunderlich FT (2013). Mechanisms of chronic JAK-STAT3-SOCS3 signaling in obesity. JAKSTAT.

[32] Münzberg H, Myers MG (2005). Molecular and anatomical determinants of central leptin resistance. Nat Neurosci.

[33] Oswal A, Yeo GS (2007). The leptin melanocortin pathway and the control of body weight: lessons from human and murine genetics. Obes Rev.

[34] Yeo GS, Heisler LK (2012). Unraveling the brain regulation of appetite: lessons from genetics. Nat Neurosci.

[35] Simpson KA, Martin NM, Bloom SR (2014). Hypothalamic regulation of appetite. Expert Rev Endocrinol Metab.

[36] Baskin DG, Breininger JF, Schwartz MW (1999). Leptin receptor mRNA identifies a subpopulation of neuropeptide Y neurons activated by fasting in rat hypothalamus. Diabetes.

[37] Henry FE, Sugino K, Tozer A, Branco T, Sternson SM (2015). Cell type-specific transcriptomics of hypothalamic energy-sensing neuron responses to weight-loss. Elife.

[38] Butler MG, Moore J, Morawiecki A, Nicolson M (1998). Comparison of leptin protein levels in Prader-Willi syndrome and control individuals. Am J Med Genet.

[39] Myers SE, Davis A, Whitman BY, Santiago JV, Landt M (2000). Leptin concentrations in Prader-Willi syndrome before and after growth hormone replacement. Clin Endocrinol (Oxf).

[40] Bueno G (2000). Serum leptin concentrations in children with Prader-Willi syndrome and non-syndromal obesity. J Pediatr Endocrinol Metab.

[41] Luquet S, Perez FA, Hnasko TS, Palmiter RD (2005). NPY/AgRP neurons are essential for feeding in adult mice but can be ablated in neonates. Science.

[42] Coppoolse ER, de Vroomen MJ, van Gennip F, Hersmus BJ, van Haaren MJ (2005). Size does matter: cre-mediated somatic deletion efficiency depends on the distance between the target lox-sites. Plant Mol Biol.

[43] Stelzer Y, Sagi I, Yanuka O, Eiges R, Benvenisty N (2014). The noncoding RNA IPW regulates the imprinted DLK1-DIO3 locus in an induced pluripotent stem cell model of Prader-Willi syndrome. Nat Genet.

[44] Mercer RE, Wevrick R (2012). Energy homeostasis in Prader-Willi syndrome: how clinical research informs studies of animal models of genetic obesity: comment on “Nutritional phases in Prader-Willi syndrome,” Miller et al., 2011. Am J Med Genet Part A, 155:1040-1049. Am J Med Genet A.

[45] Purtell L, Qi Y, Campbell L, Sainsbury A, Herzog H (2017). Adult-onset deletion of the Prader-Willi syndrome susceptibility gene Snord116 in mice results in reduced feeding and increased fat mass. Transl Pediatr.

[46] Elmquist JK, Bjørbaek C, Ahima RS, Flier JS, Saper CB (1998). Distributions of leptin receptor mRNA isoforms in the rat brain. J Comp Neurol.

[47] Reed AS, Unger EK, Olofsson LE, Piper ML, Myers MG, Xu AW (2010). Functional role of suppressor of cytokine signaling 3 upregulation in hypothalamic leptin resistance and long-term energy homeostasis. Diabetes.

[48] Elia M, Livesey G (1992). Energy expenditure and fuel selection in biological systems: the theory and practice of calculations based on indirect calorimetry and tracer methods. World Rev Nutr Diet.

[49] McMurray F (2013). Adult onset global loss of the fto gene alters body composition and metabolism in the mouse. PLoS Genet.

[50] Larder R (2017). Obesity-associated gene TMEM18 has a role in the central control of appetite and body weight regulation. Proc Natl Acad Sci U S A.

[51] Franklin KBJ, Paxinos G. *The mouse brain in stereotaxic coordinates*. 3rd ed. Amsterdam: Elsevier Academic Press; 2007.

[52] Tung YC, Ma M, Piper S, Coll A, O’Rahilly S, Yeo GS (2008). Novel leptin-regulated genes revealed by transcriptional profiling of the hypothalamic paraventricular nucleus. J Neurosci.

[53] Jovanovic Z, Tung YC, Lam BY, O’Rahilly S, Yeo GS (2010). Identification of the global transcriptomic response of the hypothalamic arcuate nucleus to fasting and leptin. J Neuroendocrinol.

[54] Adriaenssens A (2015). A transcriptome-led exploration of molecular mechanisms regulating somatostatin-producing D-cells in the gastric epithelium. Endocrinology.

[55] Adriaenssens AE (2016). Transcriptomic profiling of pancreatic alpha, beta and delta cell populations identifies delta cells as a principal target for ghrelin in mouse islets. Diabetologia.

[56] Yavari A (2016). Chronic activation of γ2 AMPK induces obesity and reduces β cell function. Cell Metab.

[57] Robinson MD, McCarthy DJ, Smyth GK (2010). edgeR: a Bioconductor package for differential expression analysis of digital gene expression data. Bioinformatics.

