# Supplementary material for: Hypothalamic loss of Snord116 recapitulates the hyperphagia of Prader-Willi syndrome
Source: J Clin Invest. 2018 Jan 29;128(3):960–9. doi: 10.1172/JCI97007 (PMC5824864; doi:10.1172/JCI97007)
Supplement: Supplemental data [file jci-128-97007-s001.pdf]

Supplementary Figure 1

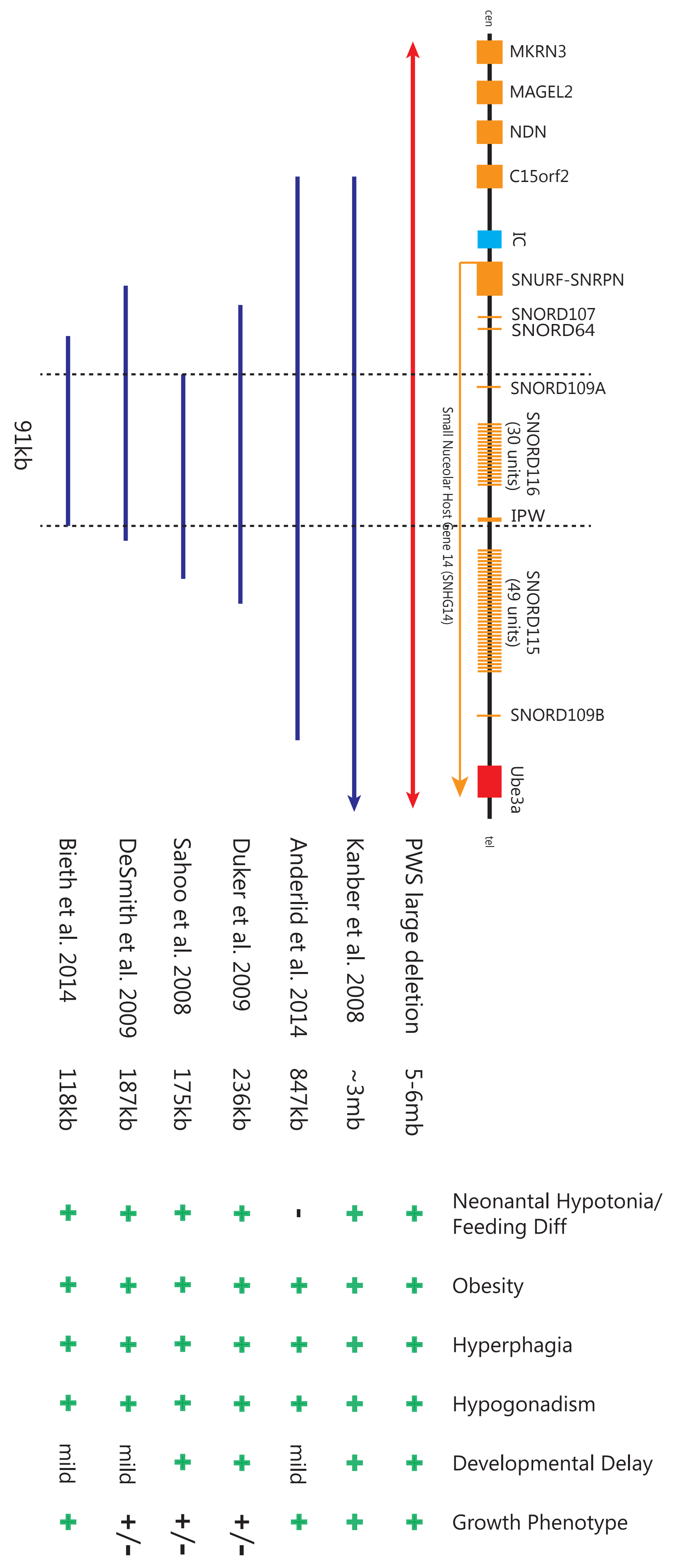

Supplementary Figure 2

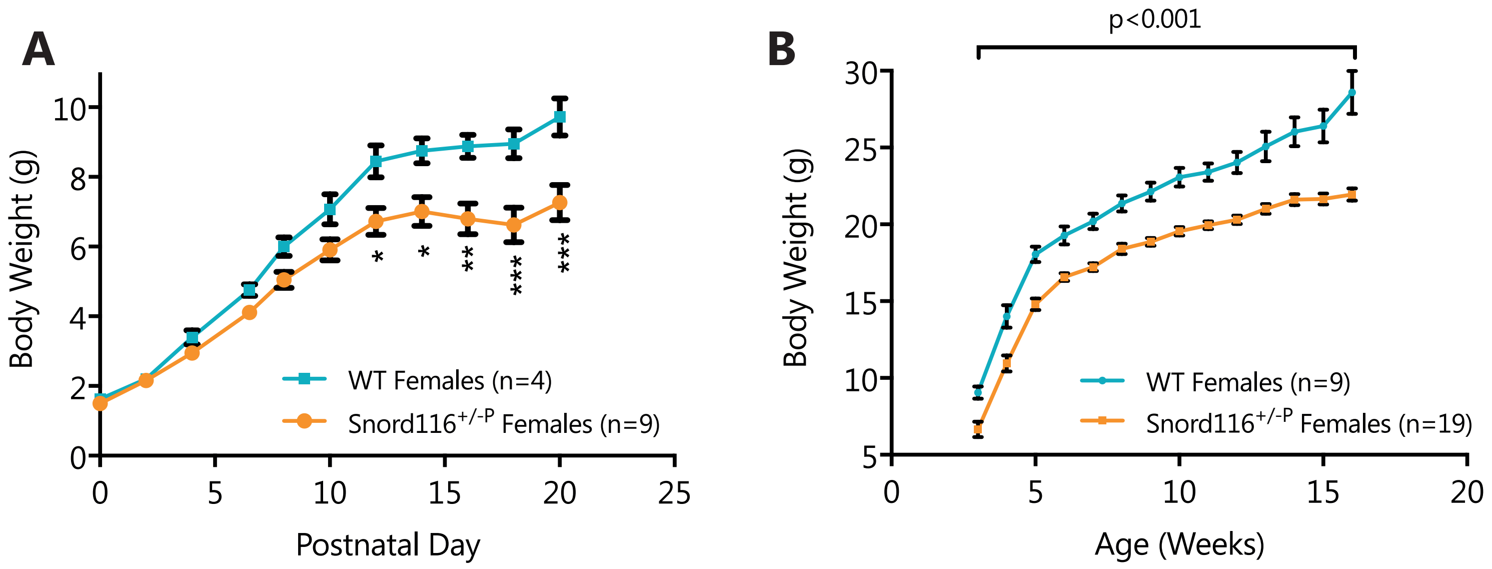

Supplementary Figure 3

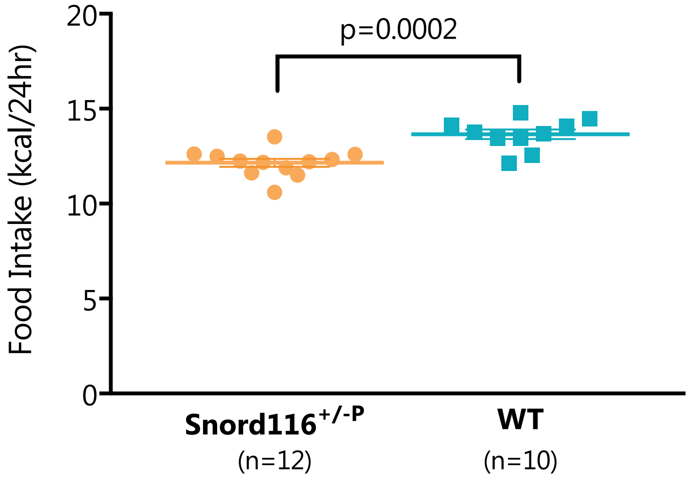

Supplementary Figure 4

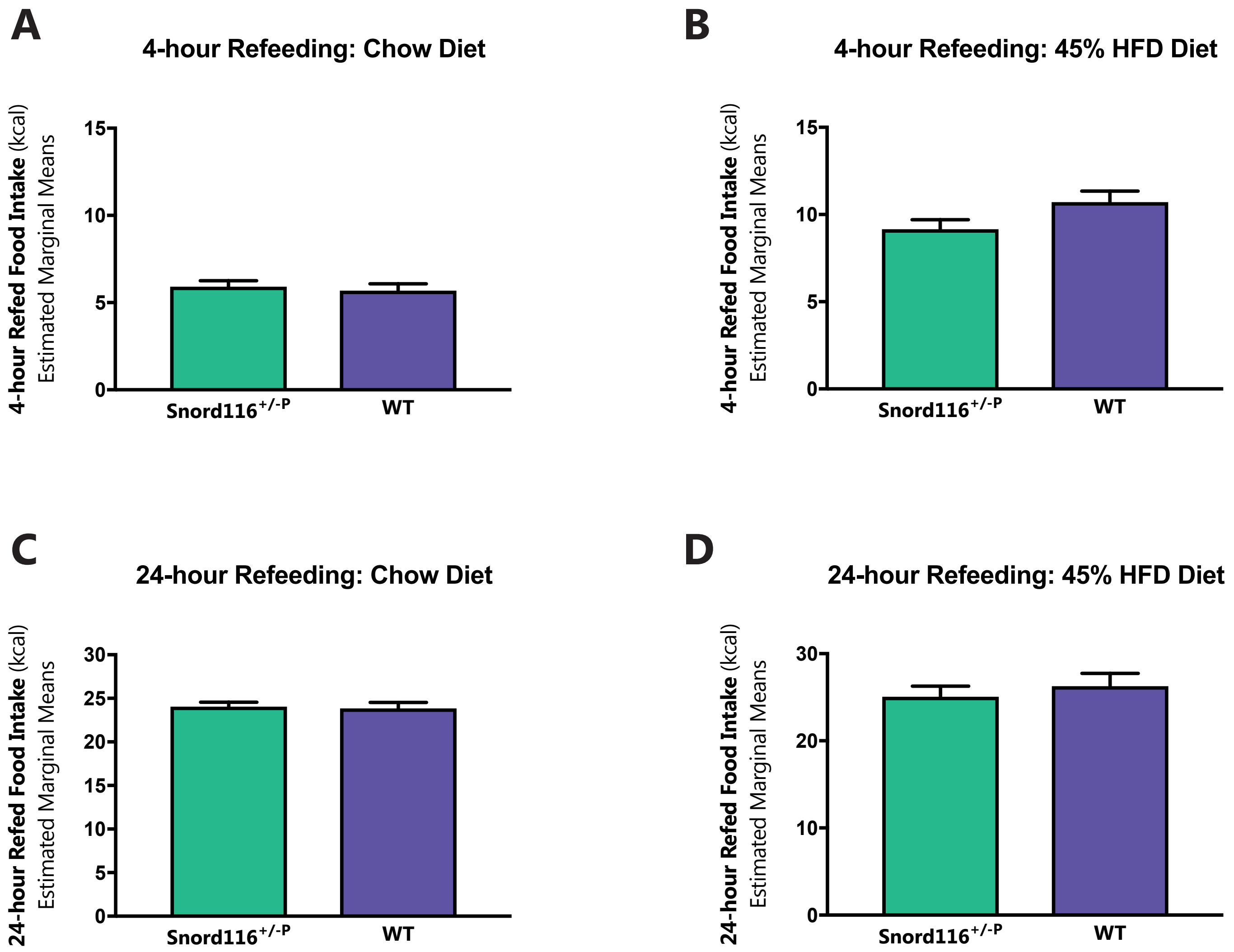

Supplementary Figure 5

A

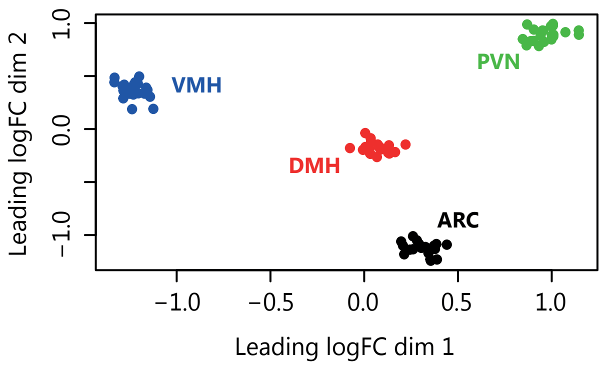

B

VMH

Gene name	Symbol	Location	logFC	p-value	FDR
Nuclear receptor subfamily 5 grp A member 1	***Nr5a1***	Nucleus	7.04	5E-49	6E-46
FEZ family zinc finger 1	***Fezf1***	n/a	5.45	4E-60	9E-57
G protein-coupled receptor 149	***Gpr149***	Plasma Membrane	4.67	2E-32	8E-30
Zinc finger protein 831	***Zfp831***	n/a	4.55	3E-29	9E-27
Neurogranin	***Nrgn***	Plasma Membrane	3.56	6E-33	3E-30
Adenylate cyclase activating polypeptide 1	***Adcyap1***	Extracellular Space	3.40	3E-34	1E-31
Dendrin	***Ddn***	Nucleus	3.26	6E-13	4E-11
Neuronal pentraxin 2	***Nptx2***	Extracellular Space	3.23	6E-18	7E-16
Ankycorbin	***Rai14***	Multiple	3.04	1E-18	2E-16
Homeodomain interacting protein kinase 4	***Hipk4***	Cytoplasm	3.00	2E-32	7E-30

ARC

Gene name	Symbol	Location	logFC	p-value	FDR
Cellular retinoic acid binding protein 1	***Crabp1***	Cytoplasm	6.79	2E-39	5E-36
Neuropeptide Y	***Npy***	Extracellular Space	5.92	7E-44	2E-40
Agouti related neuropeptide	***Agrp***	Extracellular Space	5.90	1E-38	2E-35
Proopiomelanocortin	***Pomc***	Extracellular Space	5.46	3E-79	4E-75
T-box 3	***Tbx3***	Nucleus	4.50	5E-48	3E-44
RAS guanyl releasing protein 1	***Rasgrp1***	Cytoplasm	2.89	6E-46	2E-42
Growth hormone-releasing hormone	***Ghrh***	Extracellular Space	2.76	2E-08	2E-06
Serpin family A member 3	***Serpina3n***	Extracellular Space	2.73	8E-38	1E-34
Tachykinin 2	***Tac2***	Extracellular Space	2.68	2E-05	1E-03
Leucine rich repeat containing 9	***Lrrc9***	n/a	2.59	2E-30	4E-27

PVN

Gene name	Symbol	Location	logFC	p-value	FDR
Arginine vasopressin	***Avp***	Extracellular space	7.68	1E-80	6E-77
Oxytocin/neurophysin I prepropeptide	***Oxt***	Extracellular Space	7.32	5E-83	4E-79
Urocortin 3	***Ucn3***	Extracellular Space	5.86	7E-17	2E-14
Forkhead box B2	***Foxb2***	Nucleus	5.52	7E-14	1E-11
FEZ family zinc finger 2	***Fezf2***	Nucleus	5.31	1E-18	4E-16
PR domain 8	***Prdm8***	Nucleus	5.27	1E-22	6E-20
UNC homeobox	***Uncx***	n/a	5.17	2E-40	3E-37
Single-minded family transcription factor 1	***Sim1***	Nucleus	5.12	9E-35	2E-31
Corticotropin releasing hormone	***Crh***	Extracellular Space	5.03	2E-26	2E-23
Death associated protein-like 1	***Dapl1***	n/a	4.85	1E-07	6E-06

DMH

Gene name	Symbol	Location	logFC	p-value	FDR
hypocretin (orexin) neuropeptide precursor	***Hcrt***	Extracellular Space	8.25	2E-42	2E-38
neuropeptide VF precursor	***Npvf***	Extracellular Space	7.70	2E-79	3E-75
pro-melanin concentrating hormone	***Pmch***	Extracellular Space	5.59	2E-24	7E-21
PARP1 binding protein	***Parpbp***	Nucleus	5.58	5E-28	2E-24
G protein-coupled receptor 50	***Gpr50***	Plasma Membrane	3.95	6E-13	7E-10
histidine decarboxylase	***Hdc***	Cytoplasm	2.61	8E-09	4E-06
galanin and GMAP prepropeptide	***Gal***	Extracellular space	2.31	2E-10	2E-07
Distal-Less Homeo Box 1	***Dlx1***	Nucleus	2.18	8E-05	8E-03
protein phosphatase 1 regulatory subunit 17	***Ppp1r17***	Cytoplasm	1.99	6E-15	2E-11
suppression of tumorigenicity 18, zinc finger	***St18***	Nucleus	1.96	2E-09	9E-07

Supplementary Figure 6

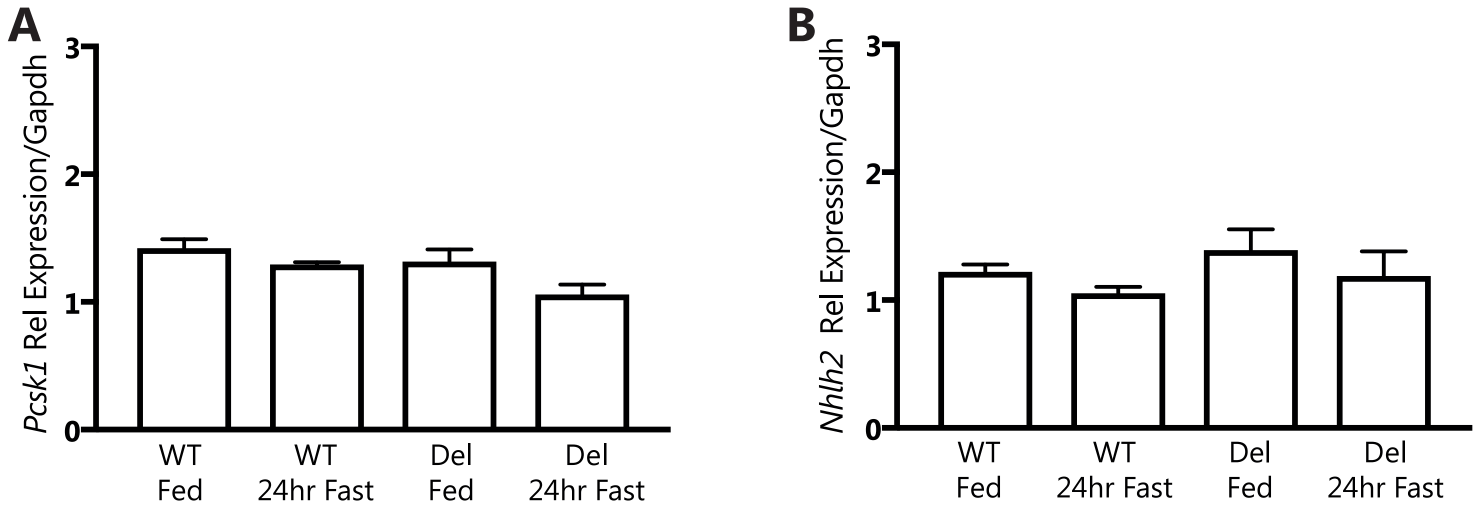

Supplementary Figure 7

A. Cre targeting with stereotaxic surgery

Images of brain sections from the mediobasal hypothalamus with Cre immunostaining (rabbit anti-Cre, 1:10,000, Novagen) visualised with DAB. Staining was used to determine “hits” or “misses” in terms of stereotaxic injection of AAV-Cre, with hits defined by the presence of Cre expression bilaterally within any portion of the mediobasal hypothalamus, from the start of the arcuate nucleus (~Bregma -1.22 through to the end of the third ventricle ~Bregma -2.70).

ID	MBH (Anterior) Cre IHC	MBH (Posterior) Cre IHC	Targeting	% original BW at 10 wks post-surgery
1	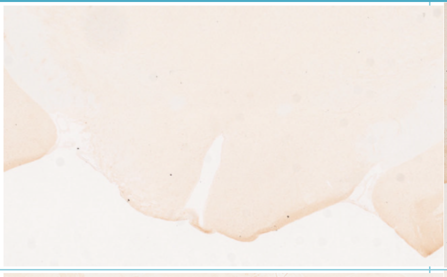	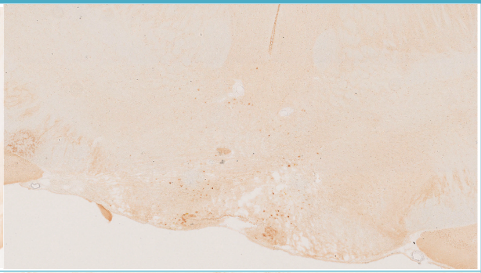	**Hit**<br>Arc	142.41
2	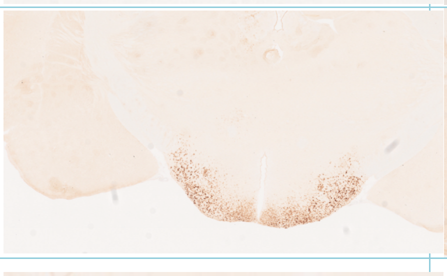	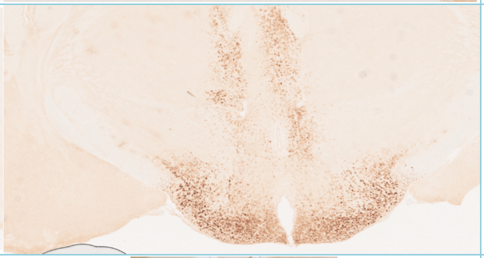	**Hit**<br>Arc<br>VMH<br>LH	114.12
3	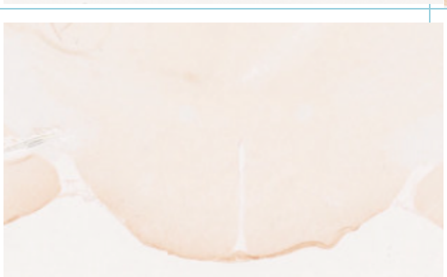	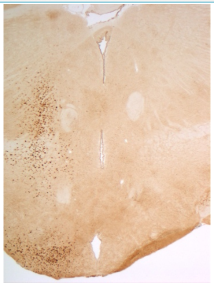	**Miss**<br>Unilateral	108.70
4	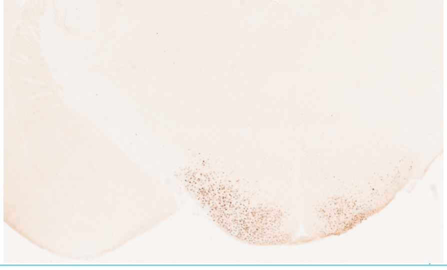	**N/A**	**Hit**<br>Arc<br>VMH<br>LH	106.14

Supplementary Figure 7

ID	MBH (Anterior) Cre IHC	MBH (Posterior) Cre IHC	Targeting	% original BW at 10 wks post-surgery
5	**N/A**	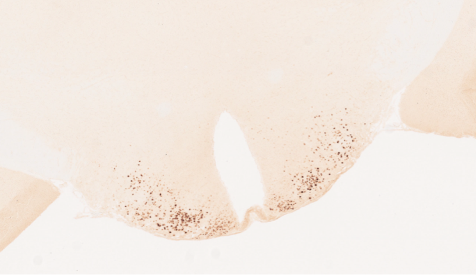	**Hit**<br>Arc<br>VMH<br>LH	150.53
6	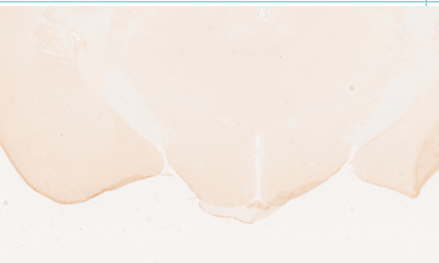	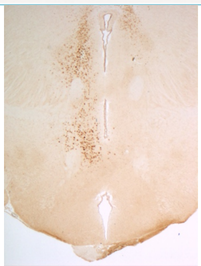	**Miss**	111.51
7	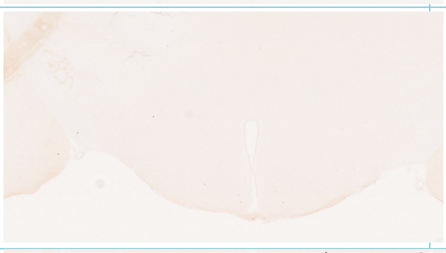	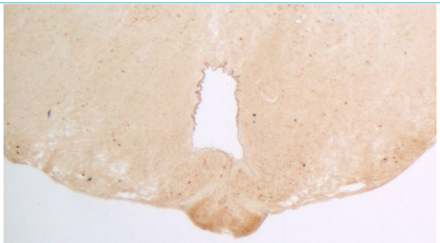	**Hit**<br>Arc	132.17
8	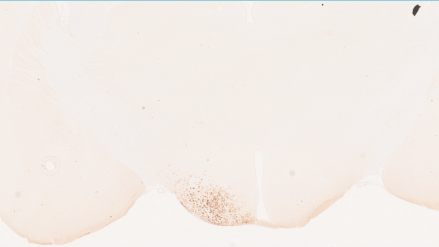	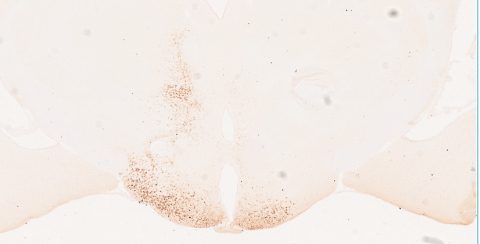	**Hit**<br>Arc<br>VMH<br>LH	113.36
9	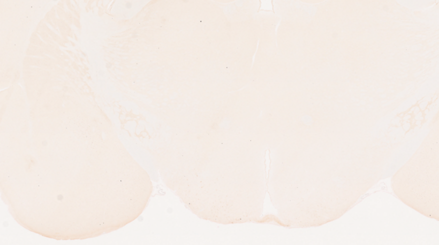	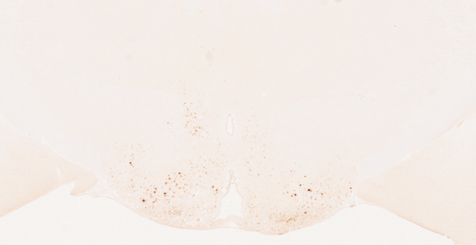	**Hit**<br>Arc<br>VMH<br>LH	109.82
10	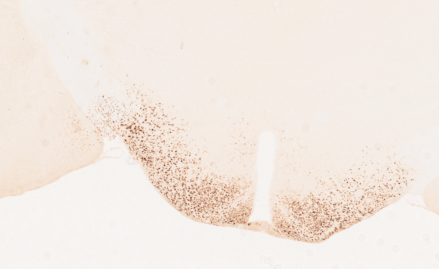	**N/A**	**Hit**<br>Arc<br>VMH<br>LH	140.48

Supplementary Figure 7

ID	MBH (Anterior) Cre IHC	MBH (Posterior) Cre IHC	Targeting	% original BW at 10 wks post-surgery
11	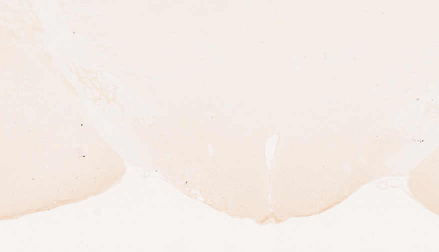	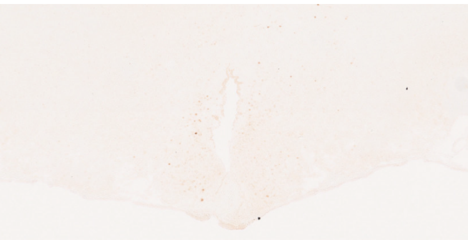	**Hit**<br>Arc	142.49
12	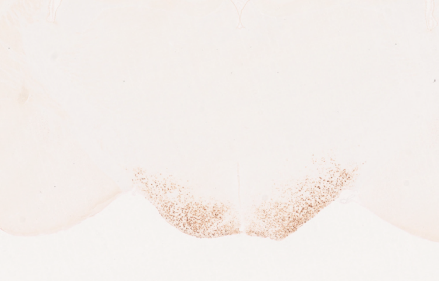	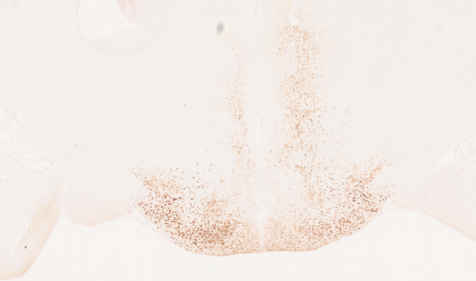	**Hit**<br>Arc<br>VMH<br>LH	124.28
13	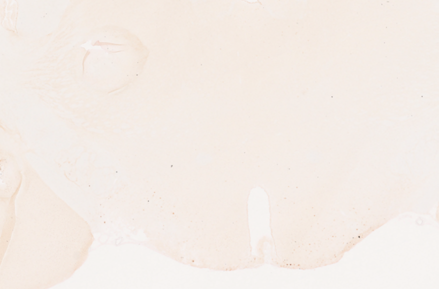	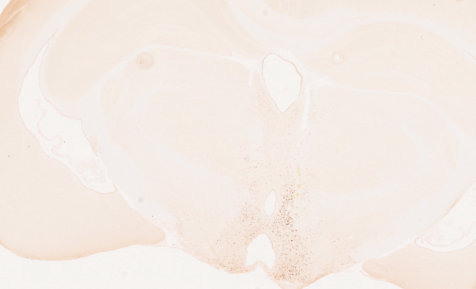	**Hit**<br>Arc	159.11
14	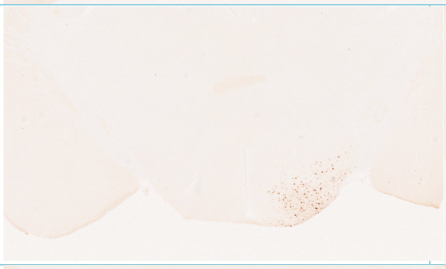	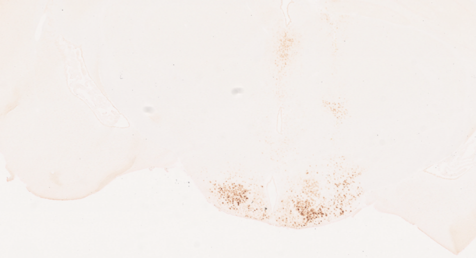	**Hit**<br>Arc<br>VMH<br>LH	126.61
15	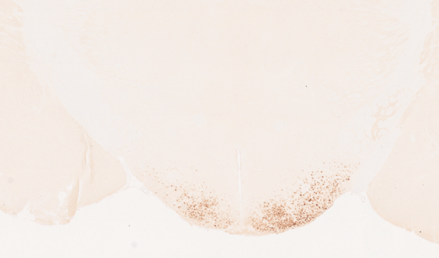	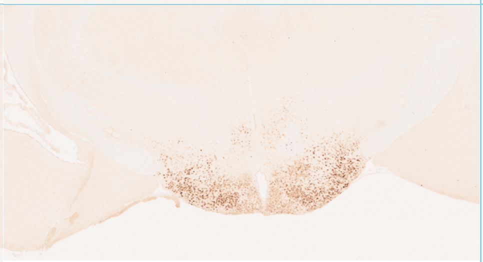	**Hit**<br>Arc<br>VMH<br>LH	114.60
16	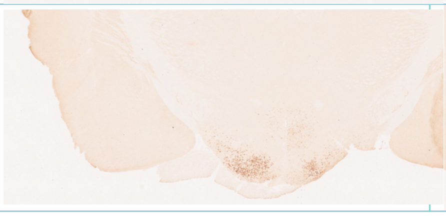	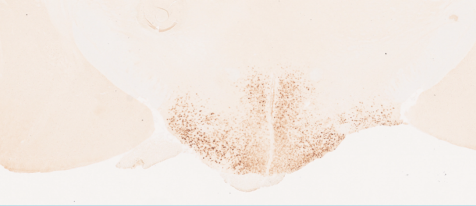	**Hit**<br>Arc<br>VMH<br>LH	107.58

ID	MBH (Anterior) Cre IHC	MBH (Posterior) Cre IHC	Targeting	% original BW at 10 wks post-surgery
17	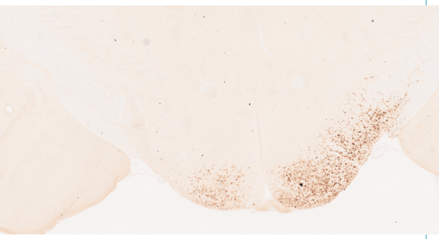	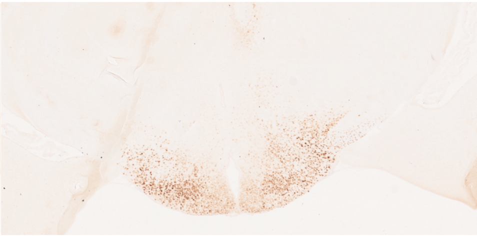	**Hit**<br>Arc<br>VMH<br>LH	107.94
18	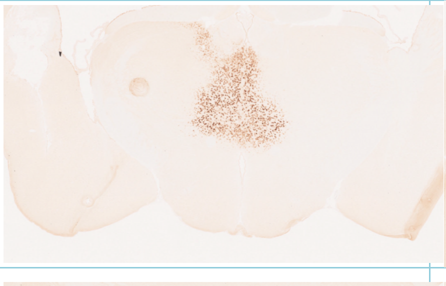	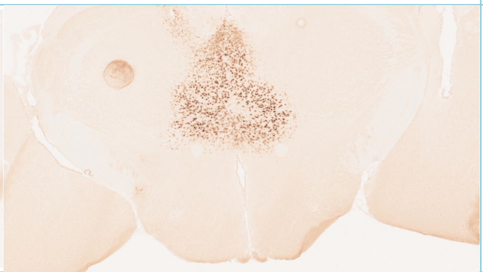	**Miss**	118.18
19	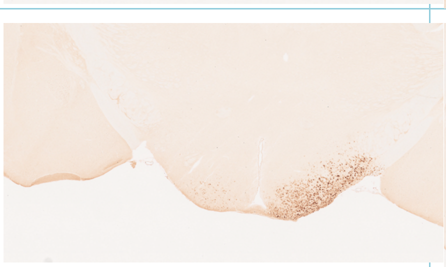	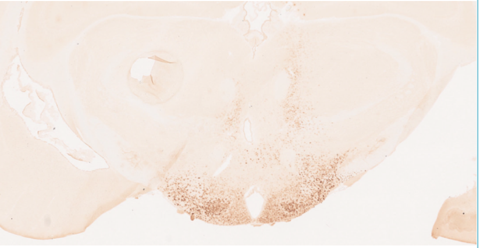	**Hit**<br>Arc<br>VMH<br>LH	123.94
20	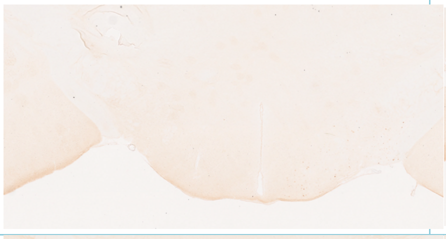	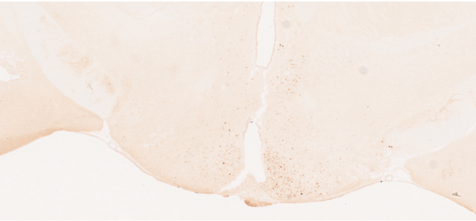	**Hit**<br>Arc	123.83
21	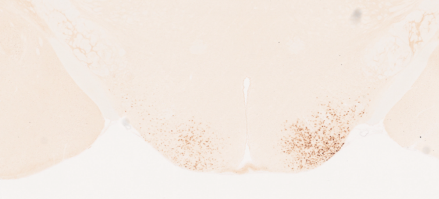	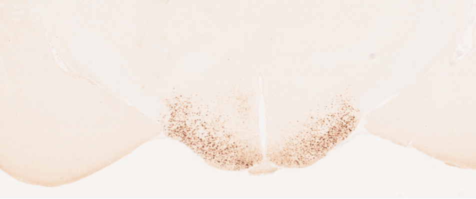	**Hit**<br>Arc<br>VMH<br>LH	124.73
22	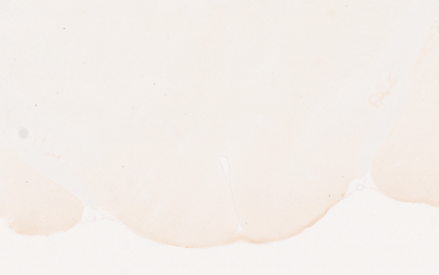	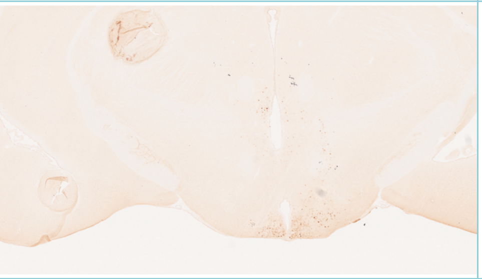	**Hit**<br>Arc	114.89

Supplementary Figure 7

ID	MBH (Anterior) Cre IHC	MBH (Posterior) Cre IHC	Targeting	% original BW at 10 wks post-surgery
23	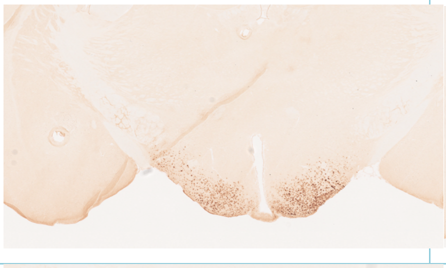	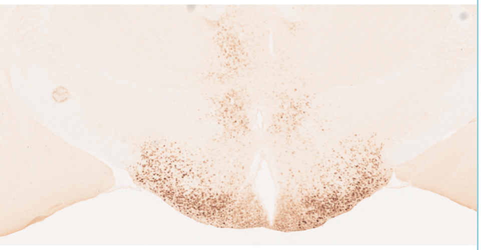	**Hit**<br>Arc<br>VMH<br>LH	109.75
24	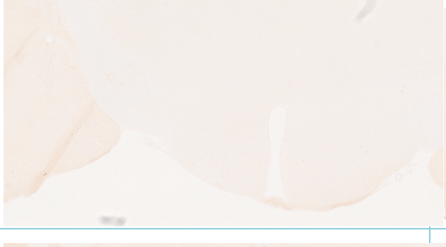	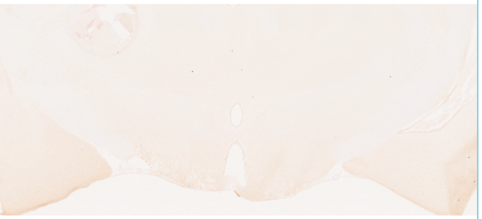	**Miss**	131.77
25	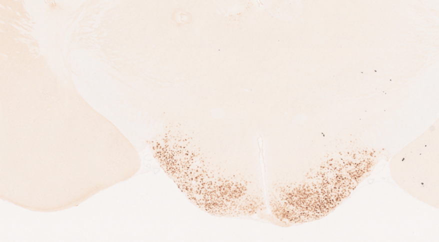	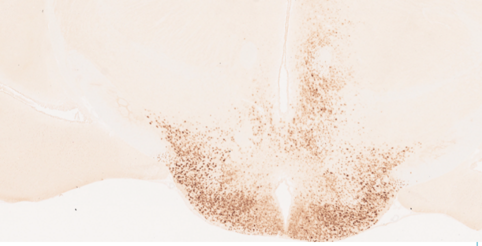	**Hit**<br>Arc<br>VMH<br>LH	108.90
26	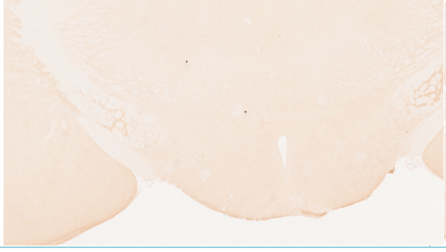	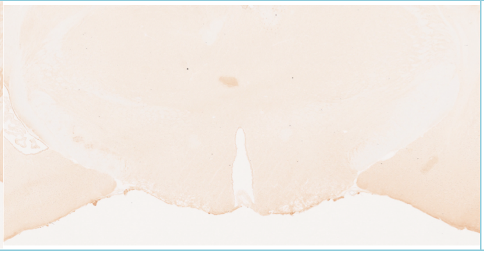	**Miss**	119.86

B. Comparison of targeting sites and final body weight

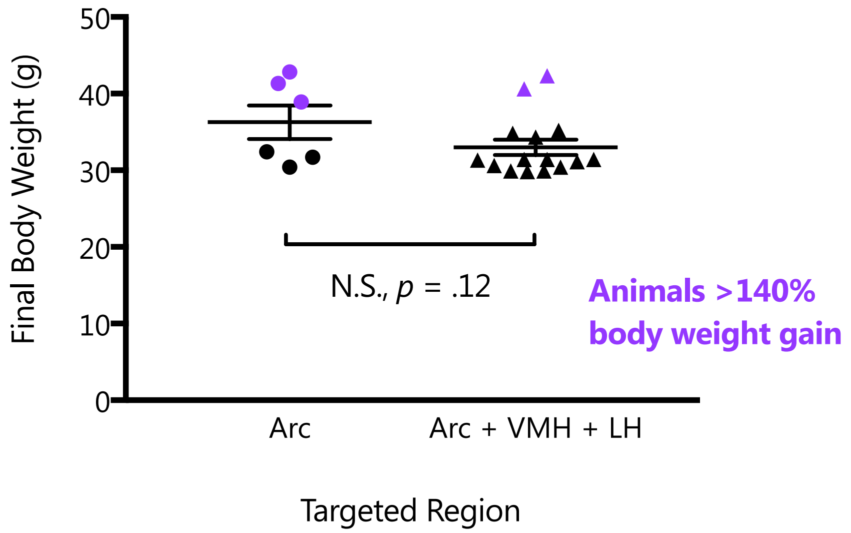

Supplementary Figure 8

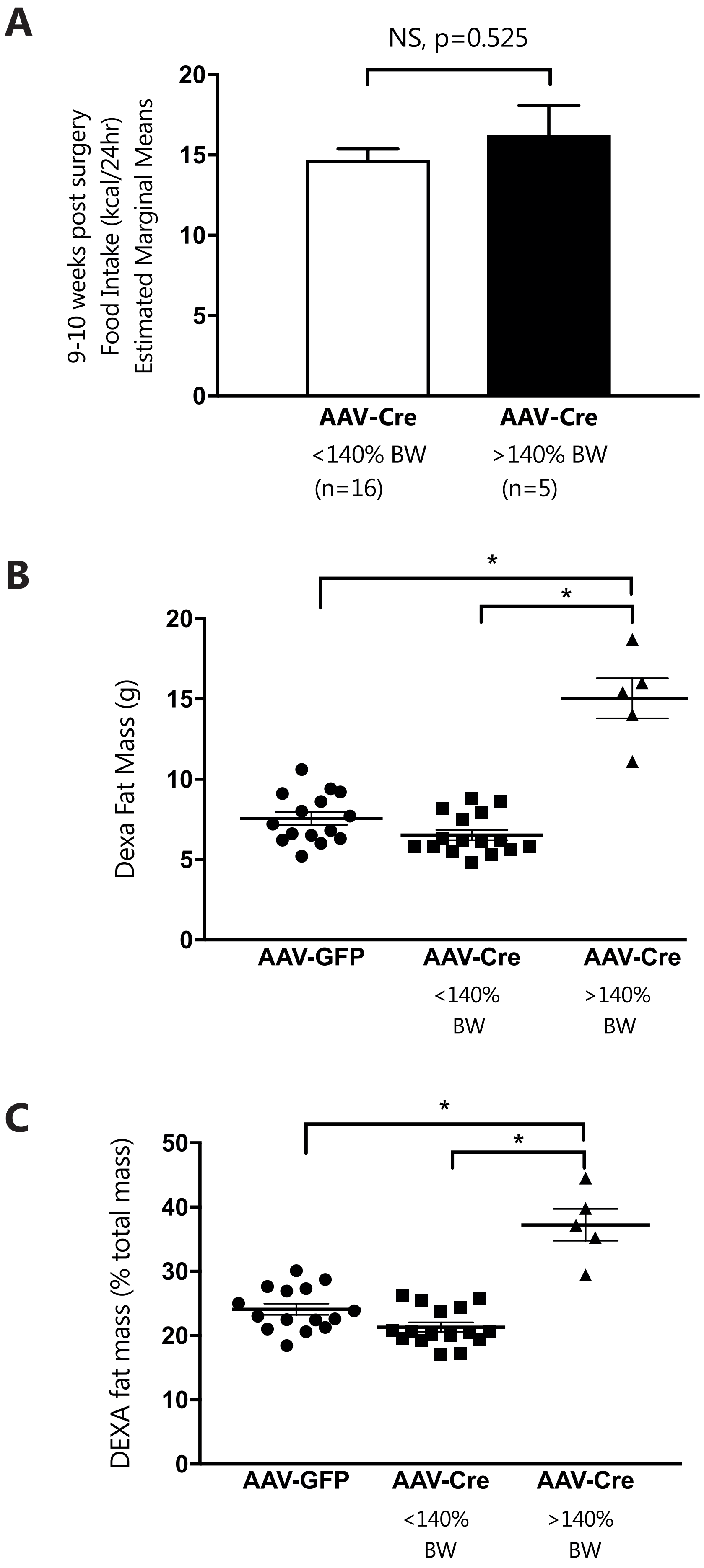

Supplementary Figure 9

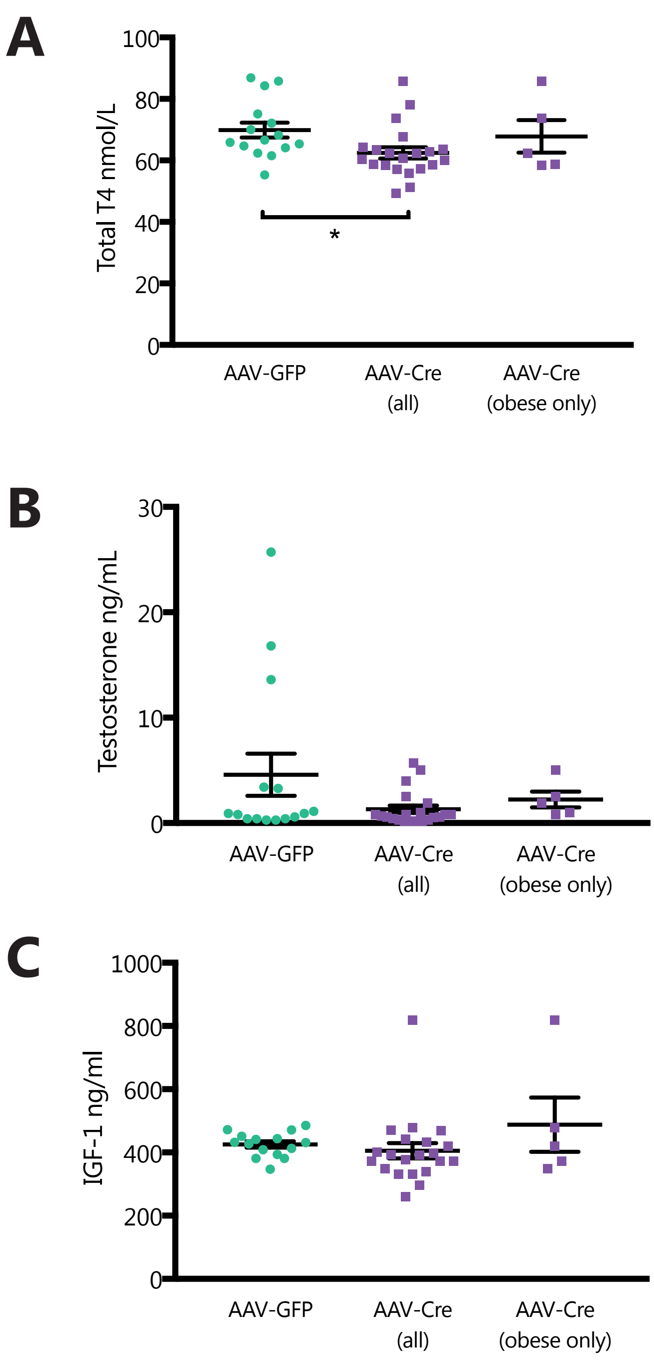

Supplementary Figure 10

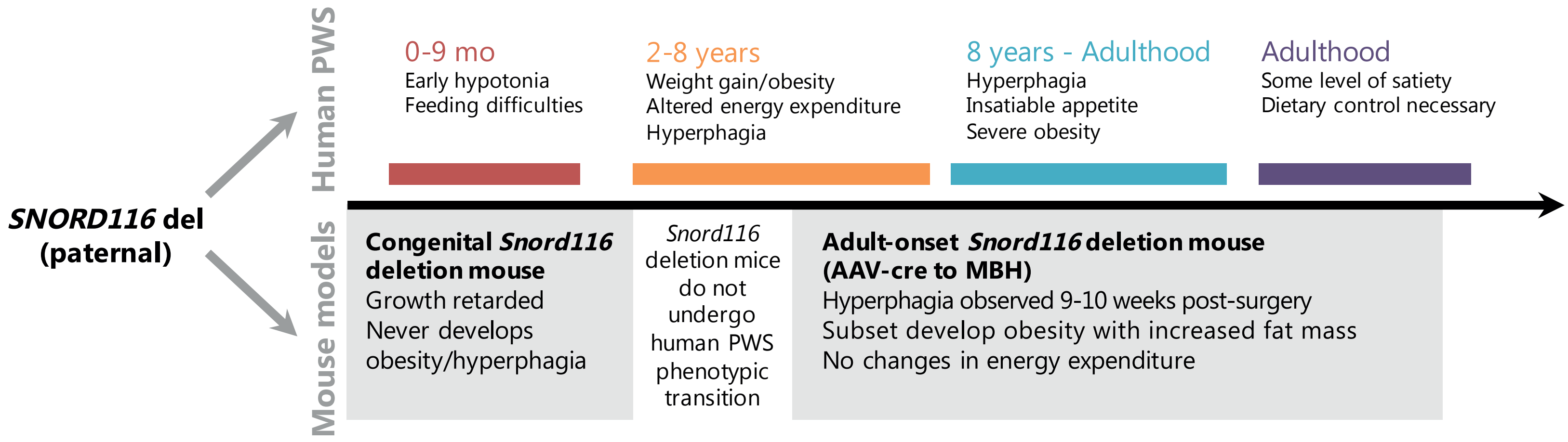

Supplementary Figure Legends

Supplementary Figure 1

***De novo* deletions encompassing *SNORD116* are associated with most major PWS clinical phenotypes.**

Summary of published small deletions encompassing the *SNORD116* cluster. (+) indicates presence of the major clinical criterion in patient with deletion; (-) absence of phenotype; (+/-) partial agreement with PWS phenotype, with Duker et al. 2009 and Sahoo et al. 2008 patients displaying normal height and the DeSmith et al. 2009 patient displaying normal-sized hands and feet in contrast to typical PWS large deletion patients.

Supplementary Figure 2

Postnatal and adult body weight is lower in female *Snord116*$^{+/-P}$ mice compared to WT mice.

(A) Postnatal body weight is lower in *Snord116*$^{+/-p}$ vs WT female mice, measured to 20 days after birth. Significant weight differences were observed from day 12 (two-way repeated measures ANOVA, ** p<0.01, *** p<0.001) **(B)** Adult body weight in *Snord116*$^{+/-p}$ female mice remains below that of WT mice, measured between 3 to 16 weeks of age, with significant effect of genotype on body weight observed throughout adulthood (two-factor mixed-design ANOVA). Data shown as mean ± SEM.

Supplementary Figure 3

Raw average daily food intake is lower in *Snord116*$^{+/-P}$ compared to WT mice.

Daily food intake (10-day average) for *Snord116*$^{+/-P}$ mice is significantly lower than that observed for WT mice (t-test, p=0.0002). Data shown as mean ± SEM.

Supplementary Figure 4

Re-feeding food intake after a 24-hour fast appears unaltered in *Snord116*$^{+/-p}$ mice either on chow or 45% high-fat diet.

Re-feeding food intake ANCOVA-corrected for body weight across genotypes, at **(A)** 4-hours post-refeeding on chow (p=0.714), **(B)** 4-hours post-refeeding on 45% high-fat diet (p=0.206, ANCOVA), **(C)** 24-hours post-refeeding on chow (p=0.856, ANCOVA), **(D)** 24-hours post-refeeding on 45% high-fat diet (p=0.643, ANCOVA). 5 WT and 6 *Snord116*$^{+/-P}$ male mice between 8-11 weeks of age were used. All data shown as mean ± SEM. HFD, high-fat diet.

Supplementary Figure 5

Laser capture microdissection yields material specific to the dissected nucleus.

(A) Hypothalamic nuclei segregate based on global transcriptomic profiles in a multi-dimensional scaling (MDS) plot of mapped reads in normalised counts per million from hypothalamic laser captured RNA samples. **(B)** Lists of the top 10 genes (by log fold change) with enriched expression in the given nucleus vs the other three captured nuclei.

Supplementary Figure 6

***Pcsk1* and *Nhlh2* expression is unaltered in murine whole hypothalamic extracts.**

Relative expression of **(A)** *Pcsk1* and **(B)** *Nhlh2* normalised to *Gapdh* is also unaltered in whole microdissected murine hypothalami (adult male mice between 7-11 months of age), as determined by relative standard curve quantitative PCR (p>0.67 for all comparisons, two-way ANOVA with Tukey's post hoc). Bars display mean ± SEM, with n=6 (WT fed), n=5 (WT fasted), n=4 (*Snord116*$^{+/-P}$ fed), n=3 (*Snord116*$^{+/-P}$ fasted).

Supplementary Figure 7

Cre targeting with stereotaxic surgery

(A) Images of brain sections from the mediobasal hypothalamus with Cre immunostaining visualised with DAB. (Note: the image in row 10 of this figure is re-used from Figure 3A) **(B)** Comparison of targeting sites and final body weights. ARC, arcuate nucleus; VMH, ventromedial hypothalamus; LH, lateral hypothalamus.

Supplementary Figure 8

Obese AAV-Cre treated mice have increased adiposity, but not food intake, compared to non-obese AAV-Cre treated mice

(A) There is no difference in food intake between the 5 obese (>140% original body weight) AAV-Cre treated mice vs the 16 non-obese (<140% original body weight) AAV-Cre treated mice (p=0.525, ANCOVA). Obese AAV-Cre mice have **(B)** increased absolute fat mass (p<0.0001, one-way ANOVA, Tukey's) and **(C)** increased percentage fat mass (p<0.0001, one-way ANOVA, Tukey's) compared to non-obese AAV-Cre mice and AAV-GFP injected mice. Bars display mean ± SEM.

Supplementary Figure 9

Serum profiling of AAV-injected mice for total thyroxine, testosterone and insulin-like growth factor 1

Serum levels of **(A)** total thyroxine (T4), 10% decrease in all AAV-Cre hits (p=0.005, Mann-Whitney test), **(B)** testosterone and **(C)** insulin-like growth factor 1 (IGF-1) in AAV-GFP controls (n=15), all AAV-Cre hits (n=21), and obese AAV-Cre hits with >140% body weight gain post-surgery (n=5). Bars display mean ± SEM.

Supplementary Figure 10

Modulating the onset of *Snord116* deletion in mouse is necessary to recapitulate early versus late stage phenotypes of human PWS.

Overview highlighting the growth and energy homeostasis phenotypes resulting from loss of paternal *Snord116* expression in human and mouse.

Supplementary Table 1

Reported phenotypes in *Snord116* deletion mouse models.

Major phenotypes related to body weight, food intake (FI) and energy expenditure (EE) reported in bold. * represent measures reported as divided by body weight. RER, respiratory exchange ratio.

Supplementary Table 2

Differentially expressed genes in laser-captured hypothalamic RNA-sequencing of AAV-Cre and AAV-GFP treated mice.

Tables of genes differentially expressed in AAV-Cre obese (>40% body weight gain post-surgery) vs AAV-GFP injected controls; AAV-Cre non-obese (<25% body weight gain post-surgery) vs AAV-GFP injected controls; and both AAV-Cre obese and non-obese vs AAV-GFP controls.
