# Supplementary material for: Hypothalamic loss of Snord116 recapitulates the hyperphagia of Prader-Willi syndrome
Source: J Clin Invest. 2018 Jan 29;128(3):960–9. doi: 10.1172/JCI97007 (PMC5824864; doi:10.1172/JCI97007)
Supplement: Supplemental Table 1 [file jci-128-97007-s002.pdf]

Supplementary Table 1

Mouse Model	Body Weight/Length/Composition	Food Intake/Energy Expenditure	Reference
**Paternal *Snord116* deletion (Brosius)**	**Growth retardation**<br>**Small body size**<br>•Growth retardation from P5<br>•Low BW into adulthood<br>•No obesity	N/A	Skryabin et al. 2007 (1)<br><br>Lassi et al. 2015 (2)
**Paternal *Snord116* deletion (Francke)**	**Growth retardation**<br>**Small body size**<br>•Growth retardation from P2; growth rate normalizes post-weaning, but weight differences remain.<br>•Smaller BW (40% smaller than WT) through adulthood<br>•Decreased body length<br>•Lower fat mass (DEXA)<br>•Low liver/stomach weight postnatally.	**↑ Food intake***<br>**↑ Energy expenditure***<br>**Resistant to HFD and calorie restriction**<br>•Ding: No difference in fast-refeeding; males 48 hours from food reintroduction have slightly higher cumulative FI<br>•Lin: Higher fast-refeeding FI from 100mins post food re-introduction	Ding et al. 2008 (3)<br><br>Lin et al. 2014 (4)
**Paternal *Snord116* deletion (Francke)**	**Small body size**<br>**All mass components ↓**<br>•Low BW through 22 weeks of age.<br>•Weight lower across fat, lean and total mass.	**No diffs in food intake (12h average)**<br>**↑ Energy expenditure***<br>•Increased day and night EE, but not independent of BW via ANCOVA.<br>•Decreased RER in light hours, normal RER cycling.	Powell et al. 2013 (5)
**Biallelic *Snord116* deletion (Francke)**	**Small body size**<br>**↓ fat mass; ↑ lean mass**<br>•Low BW through 33 weeks of age.<br>•Altered body composition in late adulthood (lower fat mass; slightly increased lean mass)<br><br><u>At 30°C:</u><br>**Low body weight remains**<br>**No difference in body length**	**↑ Food intake***<br>**↑ Energy expenditure***<br>**Resistant to HFD and calorie restriction**<br>•Increased EE (light hours) only in early adulthood.<br>•Increased fast-refeeding food intake only 48hrs from food re-introduction.<br><u>At 30°C:</u><br>**No differences in food intake***<br>**No differences in energy expenditure***	Qi et al. 2016 (6)<br><br>Qi et al. 2017 (7)
**Biallelic *Snord116* deletion (Francke) in NPY neurons**	**Small body size**<br>**↓ fat mass**<br>•Lower BW through 17 weeks of age.<br>•Shorter body length<br><u>At 30°C:</u><br>**No difference in body length**	**↑ Food intake***<br>**↑ Energy expenditure***<br>•Increased daytime RER<br><br><u>At 30°C:</u><br>**No differences in food intake***<br>**No differences in energy expenditure***<br>•Lower RER and increase physical activity	Qi et al. 2016 (6)<br><br>Qi et al. 2017 (7)
Snord116* deletion (Brosius); HPRT knock-in upstream maternal *Snord116	**“Rescue” growth delay**<br>•Slight growth delay between P7-P19.<br>•No difference in body weight compared to WT pups from 3 weeks old.	N/A	Rozhdest-vensky et al. 2016 (8)
**Tamoxifen-induced *Snord116* deletion**	**No difference in total body weight**<br>**↑ White adipose tissue**	**↓ Food intake***<br>**↓ Refeeding food intake***	Purtell et al. 2017 (9)
**Hypothalamic re-expression of *Snord116* in biallelic *Snord116* deletion**	**↓ Body weight gain**<br>**↓ Body length**<br>•No difference in fat or lean mass	<u>At room temperature:</u><br>**No difference in food intake***<br>**No difference in refeeding food intake***<br>**↑ EE***<br><u>At 30°C:</u><br>**↑Refeeding food intake at 48 hours***<br>**↑ EE***	Qi et al. 2017 (10)

Supplementary References

1. Skryabin BV et al. Deletion of the MBII-85 snoRNA gene cluster in mice results in postnatal growth retardation. *PLoS Genet.* 2007;3(12):e235.

2. Lassi G et al. Deletion of the Snord116/SNORD116 Alters Sleep in Mice and Patients with Prader-Willi Syndrome. *Sleep* 2016;39(3):637–644.

3. Ding F et al. SnoRNA Snord116 (Pwcr1/MBII-85) deletion causes growth deficiency and hyperphagia in mice. *PLoS ONE* 2008;3(3):e1709.

4. Lin D et al. Abnormal response to the anorexic effect of GHS-R inhibitors and exenatide in male Snord116 deletion mouse model for Prader-Willi syndrome. *Endocrinology* 2014;155(7):2355–2362.

5. Powell WT et al. A Prader-Willi locus lncRNA cloud modulates diurnal genes and energy expenditure. *Hum. Mol. Genet.* 2013;22(21):4318–4328.

6. Qi Y et al. Snord116 is critical in the regulation of food intake and body weight. *Sci Rep* 2016;6:18614.

7. Qi Y et al. Ambient temperature modulates the effects of the Prader-Willi syndrome candidate gene Snord116 on energy homeostasis. *Neuropeptides* 2017;61:87–93.

8. Rozhdestvensky TS et al. Maternal transcription of non-protein coding RNAs from the PWS-critical region rescues growth retardation in mice. *Sci Rep* 2016;6:20398.

9. Purtell L et al. Adult-onset deletion of the Prader-Willi syndrome susceptibility gene Snord116 in mice results in reduced feeding and increased fat mass. *Transl Pediatr* 2017;6(2):88–97.

10. Qi Y et al. Hypothalamus specific re-introduction of Snord116 into otherwise Snord116 deficient mice increased energy expenditure. *Journal of Neuroendocrinology* [published online ahead of print: January 17, 2017]; doi:10.1111/jne.12457
